# Molecular Modeling and Analysis of Cannabinoid and Cannabinoid-like Molecules Combining K-Means Clustering with Pearson Correlation and PCA

**DOI:** 10.3390/ijms262311520

**Published:** 2025-11-27

**Authors:** Rafael Campos Vieira, Érica C. M. Nascimento, João B. L. Martins

**Affiliations:** 1 Department of Pharmacy, Faculty of Health Sciences, University of Brasilia, Brasilia 70910-900, DF, Brazil; rafaelcamposunb@gmail.com; 2 Laboratory of Computational Chemistry, Institute of Chemistry, University of Brasilia, Brasilia 70910-900, DF, Brazil; ericacristinamoreno@gmail.com

**Keywords:** cannabinoids, Alzheimer’s diseases, Pearson correlation, K-means, electronic structure, machine learning

## Abstract

More recently, cannabinoid molecules have been widely studied for their potential to treat various diseases. We used a multidisciplinary approach, combining molecular docking and machine learning tools, to identify cannabinoid-based molecules as potential acetylcholinesterase inhibitors. We brought together molecules from the classes of cannabinoids, stilbenoids, isoflavones, and other natural products, along with their electronic structure and absorption, distribution, metabolism, excretion and tolerable toxicity (ADMET) data. A novel machine learning framework (MolSimEx, Molecular Similarity Explorer) combining K-means clustering, Pearson correlation, and principal component analysis was developed to address the similarities of these groups. From the dataset, 30 molecules were selected based on docking scores below −11 kcal/mol. The K-means clustering yielded high classification accuracy on the dataset, correctly grouping the cannabinoid analogues. Additionally, these analogues clustered with classical acetylcholinesterase inhibitors such as huprine-X, huprine-W, and donepezil when considering ADMET and electronic descriptor data. Radulanin J showed the highest correlation (0.41) with donepezil’s profile, suggesting the potential of cannabinoid-derived compounds as acetylcholinesterase inhibitors.

## 1. Introduction

The medicinal use of *Cannabis sativa* L. dates back to ancient Chinese and Egyptian civilizations [1], with many compounds (more than 500) divided into flavonoids, terpenoids, and phytocannabinoids [2]. Δ-9-tetrahydrocannabinol (THC) and cannabidiol (CBD) are the most abundant natural molecules produced by cannabis [3]. Additionally, the plant produces more than 120 derivatives, generally called minor cannabinoids, in small quantities [4,5]. Recent research has shown potential antinociceptive [6], anti-inflammatory [7], and dermatological [8] activities of minor cannabinoids and other cannabinoid analogs, called cannabinoid-like molecules.

Cannabinoid-like molecules are described as compounds with cannabimimetic properties. These molecules are found in diverse plants and may share structural similarities with cannabinoids and minor cannabinoids. Examples include desmodianones and machaeriols [4].

An important field in medicinal cannabis research is the drug design for neurodegenerative diseases such as multiple sclerosis, Parkinson’s, and Alzheimer’s [9,10,11]. The multitarget effect of cannabinoid molecules is increasingly relevant for the design of novel medicines for neurodegenerative diseases. For example, in Alzheimer’s disease, some of these compounds can competitively inhibit the human acetylcholinesterase enzyme (hAChE) and bind endocannabinoid receptors, producing a neuroprotective effect [12,13,14,15]. Consequently, theoretical studies of the electronic structure of these compounds could provide insights into their reactivity, ligand–receptor interactions, and therapeutic potential [16]. By using machine learning and data mining tools that combine molecular and electronic structure properties, it is possible to develop new medicines affordably.

Machine learning (ML) methods aim to minimize the cost function, meaning they reduce the difference between the variable predicted by the model and the observed variable [17]. Deep learning (DL) methods are machine learning algorithms that involve constructing so-called deep artificial neural networks, which have several applications, such as classifying molecules, generating new molecules, and predicting physicochemical properties [18,19]. For instance, in computational biology, the successful development of the AlphaFold algorithm has enabled accurate prediction of protein structures [20]. Additionally, in organic synthesis, ML algorithms have been developed to predict more favorable routes in retrosynthetic reactions [21].

Machine learning methods are widely used in drug design [22,23,24], encompassing the discovery of new potential drugs, the prediction of biological activity, and the determination of molecular descriptors with significant biological activity. These ML models may take inputs such as in-line molecular simplifications, e.g., simplified molecular-input line-entry system (SMILES) notation, pharmacokinetic property descriptors (e.g., absorption, distribution, metabolism, excretion, and toxicity—ADMET), and electronic structure descriptors (e.g., HOMO and LUMO energies, gap values, and atomic charges). It is important to normalize or scale the data to ensure the accuracy and integrity of ML models [25], especially when the input data have different measurement units. In most cases, the input data present considerably different units of measurement, as mentioned in the examples above.

We used classical AChEIs with well-established clinical profiles, including tacrine (THA), the first FDA-approved AChEI [26], despite its hepatotoxicity concerns; huperzine A (HUP), a natural alkaloid from *Huperzia serrata* with neuroprotective properties [27]; huprine X (HUX) and huprine W (HUW), synthetic hybrids combining structural features of tacrine and huperzine A [28,29]; galantamine (GNT), a natural phenanthrene alkaloid that also modulates nicotinic receptors [30]; physostigmine (PHY), a reversible carbamate hAChE inhibitor derived from *Physostigma venenosum* [31]; and ladostigil (LADO), a dual hAChE-monoamine oxidase inhibitor with neuroprotective properties [32]. Additionally, we included the natural hAChE substrate acetylcholine (ACh) and dopamine (DOP) as neurochemical references.

Donepezil (DNP) was chosen as a key reference compound because of its favorable pharmacokinetic profile and current status as a first-line treatment for Alzheimer’s disease. We employed two machine learning algorithms to analyze molecular property relationships among cannabinoids, cannabinoid-like compounds, and these classical AChEIs: similarity matrix and K-means clustering. We implemented these algorithms specifically for the data structure to identify patterns within molecules of the same class and between different classes. Additionally, we used principal component analysis (PCA) to identify the molecular descriptors that most significantly contributed to the pharmacophoric profiles of the studied molecules. All algorithms were integrated into a Python package version 3.9 called MolSimEx (short for Molecular Similarity Explorer).

## 2. Results

The frontier molecular orbital values and electronic descriptors are provided in Tables S3 and S4 of the Supplementary Material. The molecular orbital energy values of cannabinoids and cannabinoid-like molecules did not significantly differ between the maximum and minimum values of each orbital of the studied molecules. The most significant difference was found in the LUMO orbital (1.70 eV) between D9CT (−0.17 eV) and DMDD (−1.87 eV), while the smallest difference was found in the LUMO+4 orbital (0.49 eV), between D9CT (0.47 eV) and RDLH (−0.02 eV), excluding the energy values of classical drugs. The slight difference between the highest and lowest values for the HOMO-4 orbital (1.45 eV) between DMDE (−6.68 eV) and CNBA (−8.13 eV) is similar to the difference observed for the LUMO orbital, reflecting the similar electronic distribution of the molecules. Owing to the electronic similarities among cannabinoids, distinguishing classes without machine learning tools is challenging. This topic is particularly important, as ES descriptors are relevant in modeling the pharmacophoric profile and defining drug classes [33].

**Figure 1:**
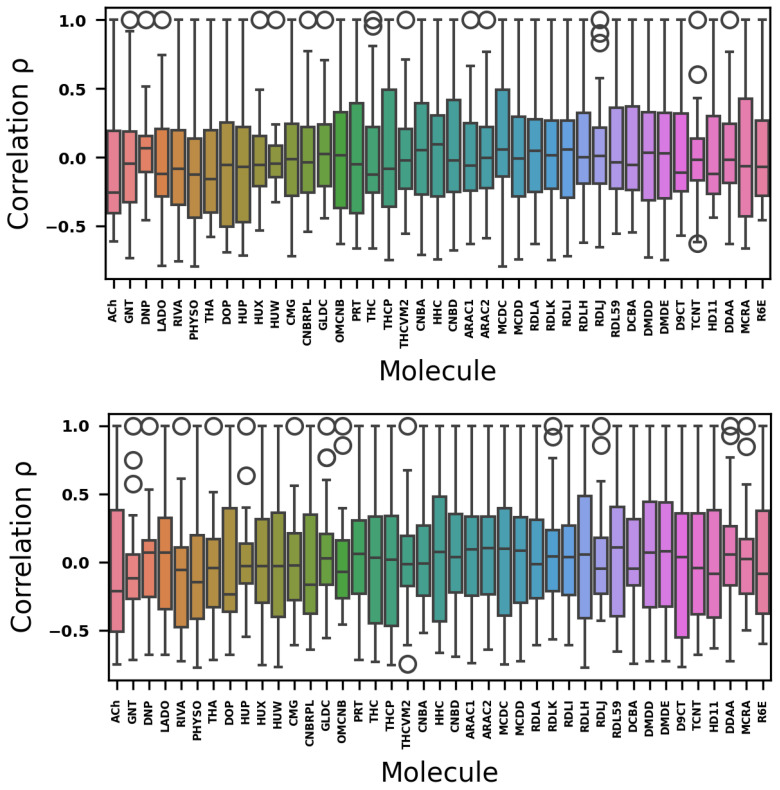
Boxplot with ADMET (**top**) and electronic structure data (**bottom**). Colors are used to facilitate the visual differentiation of molecules.

The general descriptors and ADMET data (Tables S5 and S6) are highly dispersed, as shown in a similarity matrix depicting the ES and ADMET property vectors for each molecule, expressed as percentiles. The similarity matrix is shown in the Supplementary Material (Figures S1–S3). Figure 1 shows the correlation between the ADMET and ES data spaces across the set of molecules.

When the data are normalized (Figure 1), outliers can be interpreted as having significant positive or negative correlations (ρ) for molecules with the highest and lowest property vectors. Therefore, unique outliers above the upper limits, such as those observed in the DNP for ADMET data and in the THA for ES data, correspond to the autocorrelation values of these molecules in the similarity matrix. It is worth noting that HUW exhibits a substantial increase in correlation within the ES data space compared with the ADMET data space. Conversely, molecules such as GNT and MCRA show lower correlation in the ES data space than in the ADMET data space. In contrast, others, such as RDLJ, maintain a nearly constant correlation with both ADMET and ES data.

Figure S6 displays the results of applying the K-means algorithm to the ADMET and ES data separately, using a correlation matrix, $M_{\mathrm{corr}}$, as the data space, with k = 5. The feature space illustrated in the image is based on the first row (*x*-axis) and the last row (*y*-axis) of the dataset (Tables S3–S6 of the Supplementary Material).

A comparative analysis between ADMET and ES data, using k = 5, revealed significant differences (Figures S6 and S7 of the Supplementary Material) in the positioning of specific molecules within the data space, suggesting that similarities between molecules may change depending on the data type considered. For instance, OMCNB is positioned close to DNP in the ADMET data space, whereas it is close to THA in the ES data space. The ADMET data showed an even distribution of cluster members, whereas the ES data showed a concentration of molecules primarily in clusters 1 and 4 (Figure S6). Cluster 3 in the ES data has increased considerably, driven by the inclusion of cannabinoids such as THC, THCP, HD11, and D9CT, along with ACh. Moreover, the THA in the ES data was migrated to cluster 2. Although MCDC and CNBA remained grouped in cluster 1, other molecular groups varied between the datasets. For example, HUP clustered with cannabinoids such as THCVM2, TCNT, and CMG, as well as the classical AChEI THA, which is in cluster 2 of the ES data. At the same time, classical inhibitors HUX and HUW co-clustered with cannabinoid-like molecules DMDD and DMDE, along with the prenyl bibenzyls RDLH, RDLI, and RDHJ. These associations suggest potential targets for drug repositioning studies.

**Figure 2:**
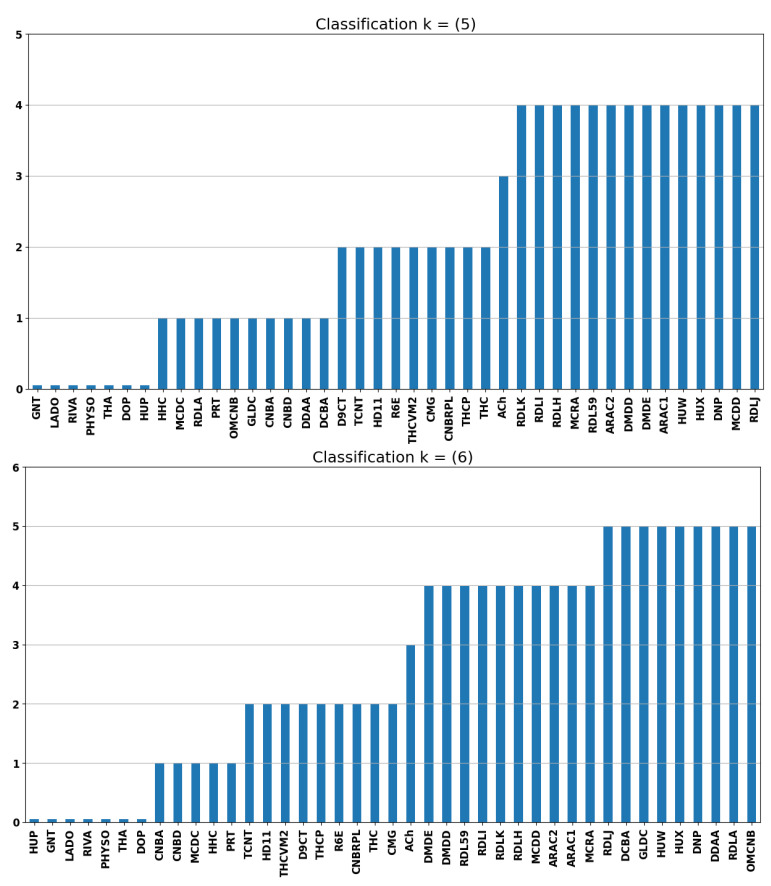
K-means ADMET and ES data with k = 5 (**top**) and k = 6 (**bottom**).

A set of molecules showed unexpected clustering patterns when the ADMET and ES data spaces were separated, with k = 5. Therefore, the next step was to use ADMET and ES data simultaneously for the K-means algorithm. For instance, separating RDLA from other prenyl bibenzyls was only possible when ADMET and ES data were combined, with k≤5, as shown in Table S7 and Figure 2. Figure S8 in the Supplementary Material displays the results of clustering using both ADMET and EE data.

As illustrated in Figure S9 of Supplementary Material, the Pearson similarity matrix has correctly grouped the classical acetylcholinesterase inhibitors (AChEIs) based on their ADMET and ES data. For example, there is a positive correlation of 0.51 between the alkaloid cholinomimetics physostigmine and galantamine. Furthermore, the synthetic compounds HUX and HUW, derived from a combination of tacrine and huperzine, also show a positive correlation of 0.57.

Table 1 shows the similarity index higher than or equal to 0.75 for the ADMET, ES, and ADMET + ES data. As expected, acetylcholine (ACh) shows no significant positive correlations with minor cannabinoids and cannabinoid analogs when analyzed with ADMET data (as illustrated in Figure S2 of Supplementary Material).

The largest vectors in loadings (Figure S6 of Supplementary Material) for the first five PCs were HOMO-4 and HOMO-3 (0.28) for PC1, LUMO+3 (0.36) for PC2, TPSA (0.47) for PC3, charge on heteroatoms and volume (0.36) for PC4, and drugscore (0.67) for PC5.

**Figure 3:**
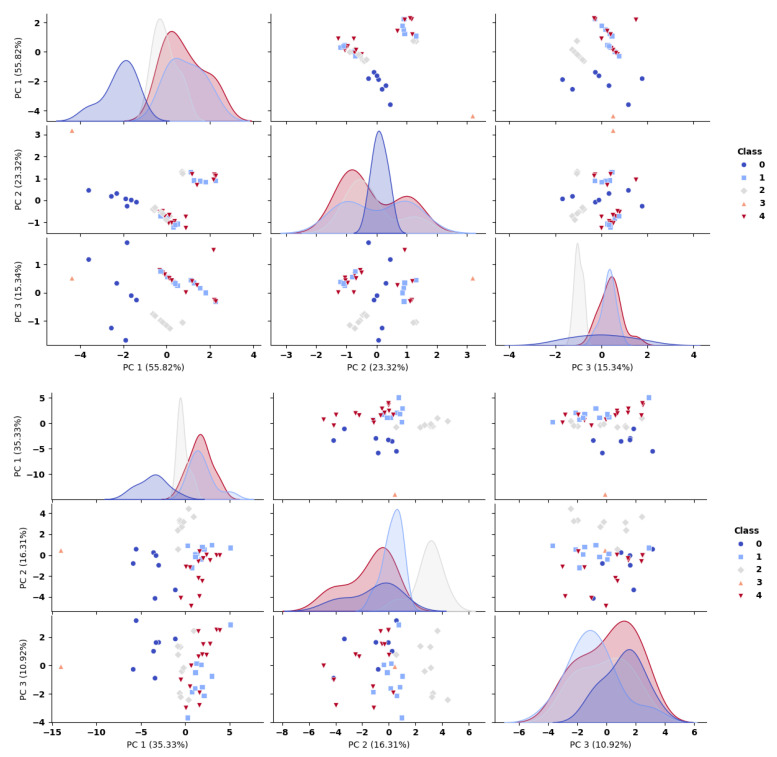
PCA scatter matrix for first 3 PCs with remaining descriptors (**top**) and with all descriptors (**bottom**) using classes (symbols and colors) predicted by k-means algorithm. The molecules in each class are presented in Table S7.

The distribution of molecules in the ADMET and ES data space can be visualized using the first three principal components (PCs) of the molecular space, as illustrated in Figure 3. This visualization uses bi-dimensional scatter plots generated from PCA, following dimensionality reduction through loading analysis. In Figure 3, each class is represented by a distinct color and symbol. Figure 3 illustrates the molecular distribution in the reduced-dimensional space of the remaining descriptors after PCA dimensionality reduction. The diagonal panels display the density distributions of each cluster projected onto individual principal components. Higher-variance principal components (particularly the first component) provide enhanced cluster separation, as evidenced by the approximately Gaussian distributions of individual clusters along these dimensions and the reduced overlap between different clusters.

The properties retained after PCA—such as molecular weight (MW(g/mol)), number of aromatic rings, blood–brain barrier (BBB) penetration (binary descriptor), and binding score (kcal/mol) (Table 2)—demonstrate a strong ability to cluster most of the molecules examined effectively.

## 3. Discussion

The Elbow and PCA methods were applied to evaluate the k value in the K-means algorithm. The elbow method was applied to the K-means algorithm, using separate ADMET and ES properties, while PCA was applied to all properties. In the PCA approach, the value of k was assumed to be equal to the number of principal components (PCs) that collectively contributed to almost all of the data variance (k = 5), as shown in Figure S4.

The elbow method is typically used to identify the ideal number of clusters in the K-means algorithm [34]. The value of k obtained by the elbow method was k=5 for ADMET and ES data, with the maximum value of the WSS being greater (>2000) for the ADMET data than for the ES data, as shown in Figure S5. Therefore, using the variance from the dimensionality reduction of the principal components (PCs) to determine k (Figure S4) is a valid approach, which is supported by the established mathematical relationship between PCA and k-means clustering, where both methods optimize similar objective functions based on data variance minimization [35]. Specifically, PCA looks for directions (principal components) that maximize variance (minimize reconstruction error). Otherwise, k-means minimizes within-cluster variance. The eigenvalue spectrum from PCA shows a natural clustering structure, as principal components with higher eigenvalues correspond to directions of greater data separability, directly informing optimal cluster number selection.

Figure 2 suggests that the clusters up to group 4 share similarities, regardless of whether k = 5 or k = 6 for the ADMET + ES data in Table S7. In contrast to the formation of these groups, ACh is isolated from the other molecules, which is significant because it serves as a functional negative control in this study. It means that ACh does not inhibit hAChE since it is the natural substrate of the enzyme. This outcome demonstrates that using combined ADMET and ES data resulted in a clustering profile that aligns more closely with the expected outcomes. Moreover, the known molecules used in the treatment of Alzheimer’s are grouped in cluster 0.

However, DNP, HUX, and HUW are grouped with RDLJ despite k = 5 or k = 6, indicating a promising possibility of RDLJ, a cyclopropanochroman derivative from *Radula javanica* with less psychoactive effects [36]. The RDLJ has the same methoxymethyl ether group also found in DNP. Another outcome is that ARAC1 and ARAC2 are grouped together with HUW, HUX, and DNP for k = 5. ARAC1 and ARAC2 are prenylated stilbenoids with the same skeleton as stilbene and other stilbenoids, which are linked to the treatment of Alzheimer’s disease using natural products [37,38].

The highest correlation value observed for Ach, cannabinoid, and cannabinoid-like molecules was 0.33 with TCNT (Figure S4 in the Supplementary Material). However, when examining the similarity matrix using ES data (Figure S3 of Supplementary Material), ACh exhibited positive correlations with THC (0.45), THCP (0.45), THCVM2 (0.40), D9CT (0.55), HD11 (0.60), and R6E (0.57), as well as an increased correlation with TCNT (0.54). Interestingly, the correlation between hAChE inhibitors (AChEIs) decreases significantly when only the ADMET data is taken into account. These differences result in a different cluster classification in the K-means algorithm, as shown in Figure S8 for ADMET + ES properties.

The similarity index shown by the ADMET + ES (Table 1) groups most similar cannabinoid and cannabinoid-like molecules, while separating GNT, PHYSO, and RIVA. However, all cannabinoid and cannabinoid-like molecules have a positive correlation, albeit small, with the classical AChEIs in the ADMET + ES data. For this set, the largest values are found for THCMV2 (0.35 with GNT), RDLH (0.37 with HUW), RDLJ (0.41 with GNT), and TCNT (0.41 with DOP). The observed clustering of cannabinoid-like compounds with classical AChEIs can be rationalized through shared pharmacodynamic and structural features that may influence acetylcholinesterase inhibition.

Structurally, both classical AChEIs and several cannabinoid derivatives possess aromatic systems and hydrogen bond acceptor/donor groups, where the presence of such groups facilitates binding to the acetylcholinesterase active site [39,40]. Furthermore, cannabinoids have demonstrated cholinergic modulation, e.g., in the direct AChE inhibition, as reported for THC [41], and the indirect cholinergic enhancement via CB1 receptor-mediated modulation of acetylcholine release [42,43]. The prenylated compounds, especially those derived from Radula species, share structural motifs with established AChEIs, including phenolic hydroxyl groups and extended conjugated systems; such groups may contribute to enzyme–substrate interactions [44]. These similarities support the computational clustering results, suggesting that the identified correlations may reflect pharmacological relationships rather than statistical artifacts.

As illustrated in Figure 3, this is particularly suitable in the apparent grouping of ACh in class 3 (depicted by the triangle symbol with a salmon color palette). Moreover, a distinct separation is observed between class 0, which contains classical inhibitors like DNP, GNT, and HUP, and both class 2, which consists of cannabinoids such as CMG, CNBRPL, and D9CT (represented by the grey diamond symbol), and class 1, which includes cannabinoid-like molecules such as DCBA, DDAA, GLDC, and OMCNB (marked by the light blue square symbol). From Figure 3, we can also see that the molecules in cluster 4 (marked by a red inverted triangle), which consist of cannabinoid-like compounds such as ARAC1, ARAC2, and DMDD, as well as the molecules in cluster 2, are closely grouped with the classic AChEIs in cluster 0. These findings emphasize the need to treat these properties as essential descriptors of molecular characteristics, highlighting their pivotal role in molecular analysis.

Although the loading analysis retained only ADMET properties in our final model (Table 2), it is worth noting that all electronic structure descriptors exhibited high loadings (Figure S10, Supplementary Material). This feature indicates that electronic descriptors also contributed significantly to the principal components, even though they were not retained in the final dimensionally reduced model.

Furthermore, the boxplot analysis (Figure 1) showed that electronic structure data produced more positive and negative outliers than ADMET descriptors, as indicated by the Pearson correlation coefficient. These outliers represent molecules that exhibited high positive or negative correlations with the reference molecule in each boxplot analysis. This suggests that electronic structure descriptors can identify molecular similarity and dissimilarity within our dataset, thereby separating cannabinoids from classical AChE inhibitors.

The complementary nature of these descriptor types is a challenge. While ADMET properties provided the most consistent dimensionality reduction (as shown by the loadings analysis), electronic descriptors exhibit a broader distribution of correlations, suggesting that both are valuable for molecular characterization.

Future studies could use these two approaches—dimensionality reduction via loading analysis and correlation coefficient variation analysis—by developing more robust search algorithms, such as simplex optimization or Monte Carlo simulations, to better capture the combined effects of both ADMET and electronic structure descriptors in molecular clustering.

## 4. Materials and Methods

### 4.1. Docking and Electronic Structure Study

We employed molecular docking as a fast, low-cost, structure-based screening to estimate the relative binding affinities of cannabinoids and cannabinoid-like molecules towards hAChE. In the present work, docking was used strictly to generate comparable docking scores as ranking criteria and as features for subsequent machine-learning models, rather than to make definitive claims about absolute affinities or detailed binding mechanisms. By standardizing the receptor conformation and docking parameters, the resulting scores provide a consistent framework for prioritization that integrates seamlessly with our data-driven pipeline.

Our method focuses on initial screening based on the canonical binding pose for this well-studied enzyme [28,45,46,47,48,49]. Therefore, this approach is a well-suited simplification because more recent computational work using molecular dynamics has shown that donepezil’s inhibitory mechanism involves multiple stable interaction modes within the active site of hAChE [45]. The molecular docking study was performed using the AutoDock4 Tools version 4.2.6 software package [50]. The bioreceptor selected was the human hAChE crystal structure, deposited in the PDB (Protein Data Bank) under the code 6O4W. The docking study protocol was applied in accordance with the literature [51].

AutoDock Vina searched for up to 1000 solutions at an exhaustiveness level of 32. The gridbox parameters were set to a width of 25.5 Å, a height of 25.5 Å, and a depth of 25 Å. The center point was located at X = 87.6, Y = 84.7, and Z = −4.7 Å. All calculations were performed using a rigid protein–ligand approach. This methodological sequence is supported by the known structural nature of classical AChE inhibitors, which are characterized by predominantly rigid molecular frameworks [52], thereby validating the use of a rigid model to approximate their primary bioactive state [53].

All water molecules of the 6O4W structure and other molecules, such as stabilizers and artifacts, were removed. The protonation states of histidine residues in the enzyme were manually configured in AutoDock Tools, based on PROPKA 3.5 software predictions [54,55]. To validate the docking protocol, a redocking study was performed using the crystallographic structure 6O4W (resolution 2.35 Å) complexed with its co-crystallized ligand E20 (DNP).

The native ligand was extracted from the binding site and subsequently redocked using the same parameters established for the virtual screening protocol. The redocking validation yielded an RMSD value of 0.26 Å between the docked pose and the crystallographic conformation, with a docking score of −11.60 kcal/mol. The low RMSD value, well below the 2 Å threshold considered acceptable for redocking protocols [56], and the docking score, proportional to experimental $K_{i}$ values for donepezil reported in the literature [46], both validated the docking protocol. While redocking supports the chosen conformer, rigid docking overlooks receptor dynamics and conserved waters, so rankings should be viewed as first-pass enrichment, most reliable for relatively rigid ligands.

Following the validation protocol, we conducted virtual screening using AutoDock Vina [57] with 253 molecules, categorized into phytocannabinoids, flavonoids, and terpenoids, against the hAChE (6O4W) enzyme (Tables S1 and S2 of Supplementary Material). The custom dataset was assembled from curated public resources (PubChem, ChEMBL, ZINC, and KNApSAcK) and based on literature on cannabis metabolites and plant-derived flavonoids/terpenoids [11,58,59]. Molecules with binding scores ≤−11 kcal/mol (89 molecules) were selected for docking pose analysis via Discovery Studio software version 20.1. We used the binding score of −11 kcal/mol, as it is close to the redocking value [60]. All the docking poses were compared to those of donepezil.

**Figure 4:**
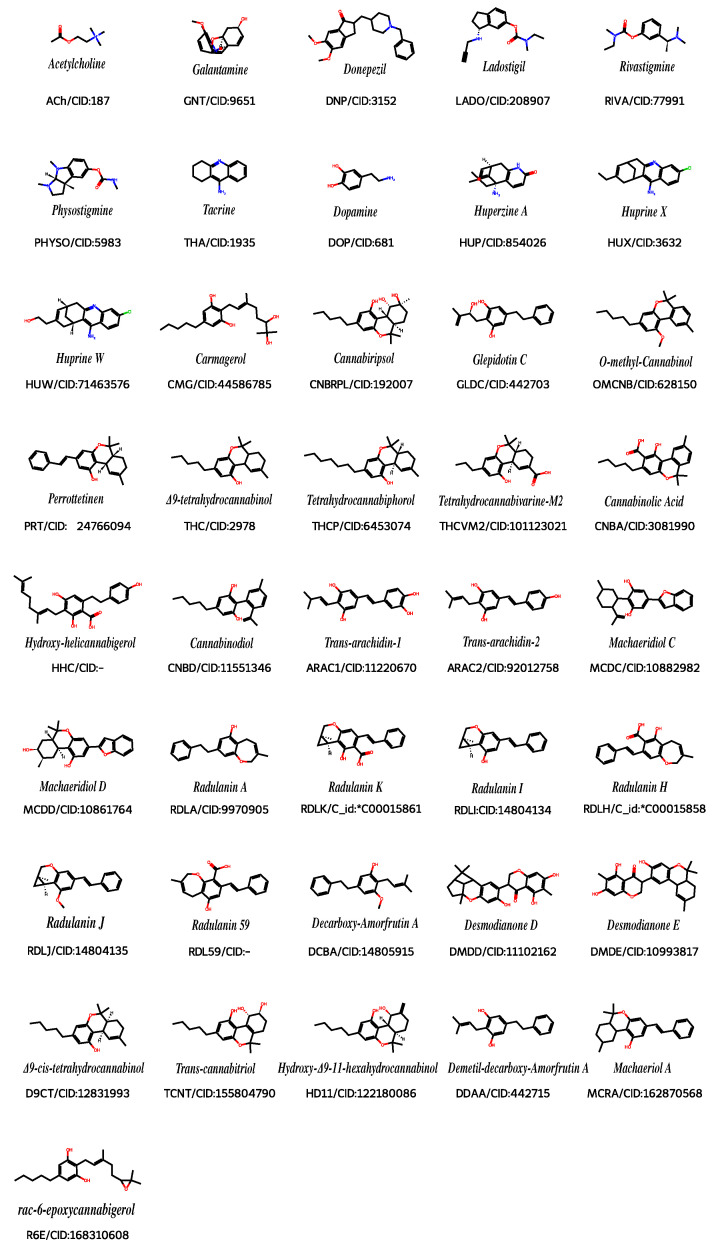
Molecules studied and the notation used. PubChem CID was also available. * C_id molecule identification found in KNApSAcK [61] metabolite database.

Molecules exhibiting interactions similar to those of donepezil, especially with the residues Trp286, Trp86, Phe338, Phe295, Ser293, and Tyr341 of the peripheral anionic site (PAS) and with residue His447 of the catalytic active site (CAS), were chosen for further analysis. Only the beta portion of the hAChE enzyme was considered in the docking protocol. The van der Waals interactions were evaluated for distances between 3 and 5 Å, whereas hydrogen bond interactions were considered for distances between 2 and 3 Å. This choice is supported by prior QM/MM molecular dynamics simulations of the AChE system [49]. Following this analysis, the remaining 30 molecules were selected for electronic structure (ES) calculations (Figure 4).

The dataset was purposely kept smaller due to the computational cost of quantum-chemical calculations, which included electronic structure descriptors at the Density Functional Theory (DFT) level. Therefore, this level of theory requires a significant computational cost per molecule. This choice prioritizes quality and physically grounded interpretability over raw scale. In practice, it is expected that these descriptors—such as dipole moment, polarizability, frontier orbital energies (HOMO/LUMO), electronic gap, global hardness/softness, partial charges, and electrostatic potentials—capture electronic aspects directly related to molecular recognition interactions and reactivity, information that is often not fully reflected by purely empirical descriptors.

The analysis of orbitals from HOMO-4 to LUMO+4 is grounded in frontier molecular orbital (FMO) theory and conceptual DFT [62,63,64]: reactivity, polarization, and charge transfer in complexation are governed not solely by a single HOMO–LUMO pair, but by multiple neighboring occupied and low-lying virtual orbitals with comparable energies and appropriate symmetry/localization [65]. Conceptual DFT demonstrates that local reactivity—via Fukui functions and local softness—is distributed across near-frontier levels rather than confined to a single orbital [66,67]. In practice, several occupied orbitals near the HOMO and low-lying virtuals near the LUMO contribute significantly, particularly when the dominant HOMO/LUMO exhibit nodes or poor localization on the binding motif [68,69]. Consequently, a symmetric ±4 window captures these contributions without excessive computational cost.

In line with these expectations, electronic descriptors have been shown to modulate the pharmacological activity of drugs, affecting the antiproliferative activity of multiple human cancer cell lines [70] and the anticoagulant effect of 17β-aminoestrogens [71].

The relevance of electronic descriptors is further highlighted in the field of AChEIs, where a QSAR analysis of tacrine-related inhibitors found that models incorporating quantum–mechanical and electronic features—such as orbital energies and charge distribution—achieved strong predictive performance for inhibitory activity [33,45,49,51,72], reinforcing the practical value of these properties for anticipating biological effects and guiding the rational design of these drugs [51,73,74].

We optimized the molecular geometries in vacuum and solvent (water) using the B3LYP [75,76,77,78] functional and the 6-311+G(d,p) basis set. The B3LYP/6-311+G(d,p) functional and basis set approach has recently been used with accurate results [51]. The implicit solvation method, based on the density model (SMD), was used (SMD) [79]. The vibrational modes, atomic charges, molar volume, and polarizability were also calculated. Vibrational modes were used to characterize the energy-minimum geometry structures. Atomic charges were calculated using the CHELPG method [80]. All electronic calculations were performed using Gaussian 16 software [81]. The descriptor distance between the most acidic hydrogens was analyzed using Chemcraft software Version 1.8, build 682 [82]. We combined similarity matrix, K-means, and PCA algorithms to analyze the results using machine learning and data mining packages such as Scikit-learn [83] and Pandas [84].

### 4.2. ADMET Data

ADMET data were obtained using the SwissADME web platform and the OSIRIS software version 2017 [85,86]. Weights were defined to obtain qualitative data on gastrointestinal (GI) absorption and blood–brain barrier (BBB) permeability.

### 4.3. MolSimEx Framework

Machine learning approaches have become increasingly popular for molecular clustering in drug discovery in recent decades [22,23]. Current computational methods for potential AChEI screening encompass various approaches, including XGBoost-based classifiers such as vScreenML, which reduce false positives in structure-based virtual screening [87]; supervised models employing Random Forest and support vector machines for compound classification [88]; web-based platforms like Tropmol that combine ML models for prediction [89]; and pharmacophore-based 3D-QSAR modeling for structural guidance [90]. These methods have shown promising results in compound identification and classification. Otherwise, they typically focus on individual prediction tasks and lack integrated frameworks for comprehensive molecular descriptor analysis and interactive clustering exploration. To address these limitations, we developed MolSimEx, an integrated computational framework that combines analytical techniques in an interactive environment designed explicitly for molecular property analysis.

The MolSimEx pipeline introduces methodological innovations that distinguish it from conventional workflows. First, we have implemented a novel distance metric called ASED (Amplified Squared Euclidean Distance). This metric enhances the separation of molecular clusters by amplifying penalties for considerable dissimilarities while preserving the relative ordering of molecular relationships. We apply this approach to molecular datasets that exhibit high variability in both similarity and dissimilarity patterns. Second, the framework integrates K-means initialization (K-means++, Bayesian, and random initialization), enabling comparisons of clustering approaches.

MolSimEx provides an interactive visualization interface that enables real-time parameter adjustment and dynamic exploration of clustering results, particularly for complex systems where standard similarity metrics may not adequately capture the cross-nature of molecular properties.

### 4.4. Similarity Matrix

Three correlation models were evaluated: Pearson, Kendall, and Spearman. Among the known similarity metrics, the choice of the Pearson correlation coefficient was motivated by its ability to capture linear relationships between molecular descriptors, which is particularly advantageous for subsequent K-means clustering analysis that benefits from broader correlation ranges. The Pearson correlation coefficient measures the correlation of two coefficients in the range of −1 to +1 [91,92]. The developed algorithm aims to generate a correlation matrix by calculating Pearson correlation coefficients between molecular descriptors. Given the set of S molecules:

(1) $$S=\left(A_{1},A_{2},A_{3},\ldots ,A_{N}\right),\;\;A_{i}=molecule\;i,$$

where each molecule is defined as a set of its descriptors

(2) $$A_{i}=\left(x_{1},x_{2},x_{3},\ldots ,\;x_{N}\right),\;\;x_{i}=descriptor\;i$$

The next step is to get a new set $S^{\prime }$ from the scaling of all descriptors:

(3) $$S^{\prime }=\left(A_{1}^{\prime },A_{2}^{\prime },A_{3}^{\prime },\ldots ,\;A_{N}^{\prime }\right),\;A_{i}^{\prime }=\left(z_{1},z_{2},\ldots ,z_{N}\right),z_{i}=\frac{x_{i}-\mu }{\sigma },$$

where μ is the mean value, and σ is the standard deviation. After scaling, the product-moment coefficients of molecules *A* and *B* are calculated.

(4) $$\rho_{A^{\prime }B^{\prime }}=\frac{cov(A^{\prime },B^{\prime })}{\sigma_{A^{\prime }}\sigma_{B^{\prime }}}\approx \frac{\sum_{i=1}^{n}\left(z_{i}^{A^{\prime }}-{\bar{z}}^{A^{\prime }}\right)\left(z_{i}^{B^{\prime }}-{\bar{z}}^{B^{\prime }}\right)}{\sqrt{\sum_{i=1}^{n}{\left(z_{i}^{A^{\prime }}-{\bar{z}}^{A^{\prime }}\right)}^{2}\sum_{i=1}^{n}{\left(z_{i}^{B^{\prime }}-{\bar{z}}^{B^{\prime }}\right)}^{2}}},$$

where ${\bar{z}}^{A^{\prime }}$ and ${\bar{z}}^{B^{\prime }}$ are the mean values of descriptors of the new scaled set of molecules $A^{\prime }$ and $B^{\prime }$. After all calculations of product-moment correlation coefficients, the similarity matrix $M_{corr}$ is generated (Algorithm 1).

(5) $$M_{\mathrm{corr}}=\left[\begin{array}{ccccc} \rho_{A^{\prime }A^{\prime }} & \rho_{A^{\prime }B^{\prime }} & \rho_{A^{\prime }C^{\prime }} & \ldots & \rho_{A^{\prime }N^{\prime }}\\ \rho_{B^{\prime }A^{\prime }} & \rho_{B^{\prime }B^{\prime }} & \rho_{B^{\prime }C^{\prime }} & \ldots & \rho_{B^{\prime }N^{\prime }}\\ \rho_{C^{\prime }A^{\prime }} & \rho_{C^{\prime }B^{\prime }} & \rho_{C^{\prime }C^{\prime }} & \ldots & \rho_{C^{\prime }N^{\prime }}\\ \vdots & \vdots & \vdots & \ddots & \vdots \\ \rho_{N^{\prime }A^{\prime }} & \rho_{N^{\prime }B^{\prime }} & \rho_{N^{\prime }C^{\prime }} & \ldots & \rho_{N^{\prime }N^{\prime }} \end{array}\right]$$

**Algorithm 1** Similarity matrix algorithm  
D←inputdata,D=structuredataobjectwithrowsandcolumns  
SD←S(D),S=Scaleroperator  
SDT←T(SD),T=Transposeoperator  
C←D(columns)  
ND←{SDT,C},ND=Newstructuredata  
$M_{\mathrm{corr}}\leftarrow MC\left(\mathrm{ND}\right),\;\;MC=Correlation\;matrix\;operator$

### 4.5. K-Means

K-means is an unsupervised machine learning algorithm in the cluster algorithms class. Stuart Lloyd of Bell Labs developed the initial version of the algorithm in 1957 [93]. The present work used the Lloyd implementation, the most common computational implementation of K-means. We employed a modified version of the traditional Euclidean norm (L2)

(6) $$L2\left(a,b\right)=\sqrt{\sum_{i=1}^{n}{\left(a_{i}-b_{i}\right)}^{2}}$$

where a and b are n-dimensional feature vectors, as the distance metric for the K-means algorithm. The variation, named ASED, is defined as:

(7) $$\mathrm{ASED}\left(a,b\right)={\left(\sum_{i=1}^{n}{\left(a_{i}-b_{i}\right)}^{2}\right)}^{2}$$

The purpose of ASED is to amplify the penalty for large distances while reducing the penalty for small distances. This approach is particularly relevant for molecular similarity assessment, as ADMET and ES properties of molecular datasets often exhibit high variability in both similarity and dissimilarity degrees. Consequently, this strategy improves navigation of the feature space induced by the Pearson-based similarity matrix, whose broader correlation spectrum expands the adequate search space for K-Means.

**Figure 5:**
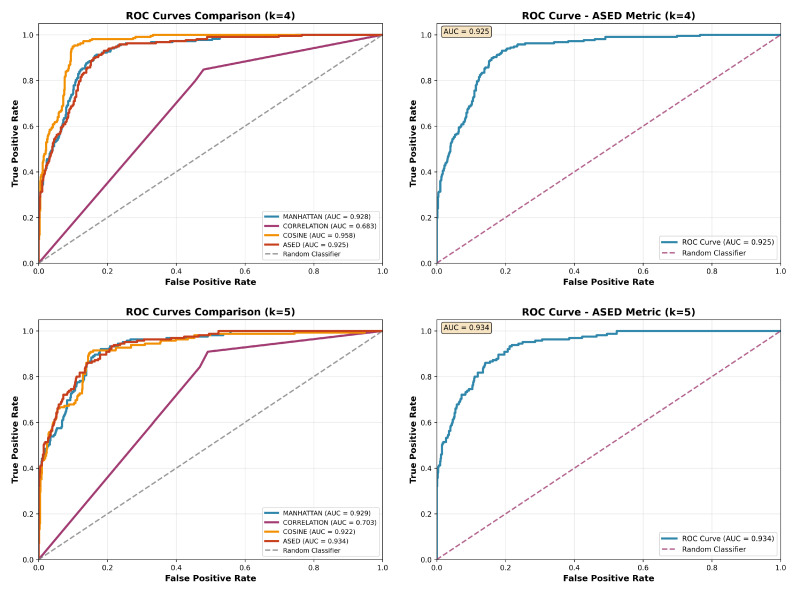
ROC curves for the ES/ADMET dataset with k = 4 (**top row**) and k = 5 (**bottom row**). The left column compares ASED (red) with Manhattan (blue), correlation (purple), cosine (yellow), and a random classifier (gray), while the right column shows ASED alone.

An additional advantage of ASED is that it constitutes a monotonic transformation of the L2 norm, which is beneficial because ROC (Receiver Operating Characteristic) analysis is invariant to monotonic transformations. This mathematical property ensures that the relative ordering of distances is preserved (see Figure 5). Notably, the ROC curves for ASED and L2 metrics are identical (fully overlapping).

We employed ROC analysis to compare ASED against other distance metrics to verify whether alternative metrics could better model the studied data. This comparative analysis allows us to identify the most appropriate distance metric for capturing the underlying structure of molecular ADMET and ES properties, thereby optimizing the clustering performance.

In drug discovery studies, K-means algorithms are currently used to identify molecular clusters and descriptors [94,95]. Other applications of the K-means algorithm are also being explored, such as identifying the best substrates for chemical reactions in molecular synthesis [96]. These applications demonstrate the broad applicability of cluster machine learning algorithms in chemistry.

Initially, the total set of data *A* is divided into k-subsets or clusters:

(8) $$A\to A^{1}\cup A^{2}\cup A^{3}\ldots A^{k}$$

Given a set of k predefined clusters, the goal of the algorithm is to obtain the centroids (c) of each cluster.

(9) $$C=(c^{1},c^{2},\ldots ,c^{k})$$

That minimizes the cost function, which for K-means is commonly named within-cluster sum of squares (WSS) or inertia, defined as the sum of the Euclidean distances between each component of the property vector, **x**, and its closest centroid. The leading information here is the mean distance among all points in a cluster.

(10) $$WSS=f\left(A,C\right)=\sum_{a=1}^{k}\sum_{x\in A^{i}}\Delta \left(x,c^{i}\right),\;\;x=\left(x_{1},x_{2},\ldots ,x_{d}\right)$$

The centroids are calculated with the expression obtained by the midpoint theorem algorithm:

(11) $$c^{i}=\frac{1}{{\left|A\right|}^{i}}\sum_{x\in A^{i}}x$$

There are many methods for determining the ideal value of *k* in the K-means algorithm [97,98,99]. In the present study, two methods were used: the elbow method, which evaluates the decay of the cost function with respect to the number of clusters (n), and PCA. The first algorithm result was applied to the K-means. This search aims to categorize classes of molecules by grouping the cannabinoid and cannabinoid-like molecules based on ES and ADMET results (Algorithm 2).
**Algorithm 2** Lloyd’s algorithm with similarity matrix as input  
$M_{\mathrm{corr}}\leftarrow input\;data$  
$Initialization:random\;centroids\;C=\{c^{1},\ldots ,c^{k}\}$  
Repeatforrange(i=1,i=2,…,i=k)$\;\;\;\;\;b\left(x\right)=argmin_{i}\left\{\Delta \left(x,c_{i}\right)\right\},\;\;\forall \;x\in M_{\mathrm{corr}}$UpdateallclusterscentroidsinC:$\;\;\;\;\;\;\;c^{i}=\frac{1}{{\left|A\right|}^{i}}\sum_{x\in A^{i}}x,\;\;\forall \;i\in \left\{1,2,\ldots ,k\right\}$  
UntilMean(clustercenters)stopchanging


## 5. Conclusions

We have identified a set of 30 molecules, out of 253 derived from phytocannabinoids, flavonoids, and terpenoids, as potential new inhibitors against the hAChE (6O4W) enzyme. We employed two machine learning algorithms to categorize the molecules: similarity matrix and K-means. These algorithms were implemented specifically for the data structure to identify patterns within molecules of the same class and between different classes. Principal component analysis was also used to find the most determining molecular descriptors.

From the K-means classification, one of the prenyl bibenzyl molecules, the cyclopropanochromane derivative radulanin J, presented the highest correlation with the AChEI profile of the drug donepezil (0.41, based on AMDET+ES data). The molecules tetrahydrocannabivarine-M2, radunalin H, radunalin J, and trans-cannabitriol showed positive correlations with the classical AChEIs in the ADMET similarity matrix and electronic structure data. The TCNT-DOP and RDLJ-DNP pairs showed a Pearson correlation coefficient of 0.41, indicating a significant correlation driven by the use of ADMET and electronic structure data.

It is important to note that, despite the promising results, particularly for radulanin J, the present computational framework includes virtual screening, requiring future experimental studies to validate the proposed insights, such as in vitro AChE inhibition assays for direct activity confirmation and $IC_{50}$ determination, cytotoxicity studies using neuronal cell lines to assess safety profiles, and blood–brain barrier permeability assays to evaluate central nervous system penetration potential.

Additionally, while our rigid docking protocol provided consistent ranking criteria for the machine learning pipeline, future studies focusing on the most promising candidates would benefit from more sophisticated computational approaches, including induced-fit docking, ensemble receptor conformations, explicit water modeling, and enhanced scoring methods, such as the molecular mechanics energies combined with the generalized Born and surface area continuum solvation (MM/GBSA) calculation, to reduce potential docking bias and provide more accurate binding predictions. These validations would provide essential pharmacological data for advancing the developed model.

Moreover, in the current MolSimEx implementation, the ASED metric is effectively equivalent to the L2 norm for clustering, yielding assignments and centroids that are indistinguishable from those of standard K-Means. Accordingly, potential advantages over the classical L2 metric have yet to emerge. Future research that improves centroid estimation and aligns the objective function with ASED may advance MolSimEx, enabling the new norm to exceed L2 in specific cases.

Although these findings are promising, they are based on a relatively narrow dataset, and broader evaluations will be needed to validate the benefits of the metrics. The increasing availability of reliable electronic structure calculations is an open field for drug discovery. Future research should broaden these analyses to include larger and more diverse chemical libraries. This research would enable the identification of even more robust and generalizable molecular similarity patterns for the rational design of new drugs.

## Figures and Tables

**Table 1:** Similarity index of ADMET, ES and ADMET + ES data.

ADMET	ES	ADMET + ES
GNT-PHYSO, GNT-HUP, RIVA-PHYSO, PHYSO-HUP, CNBRPL-TCNT, CNBRPL-HD11, OMCNB-PRT, OMCNB-THCP, OMCNB-MCRA, PRT-MCRA, THC-THCP, THC-D9CT, THC-HD11, THCP-D9CT, CNBA-MCDC, RDLA-RDLI, RDLA-RDLJ, RDLK-RDLH, RDLK-RDL59, RDLI-RDLJ, RDLH-RDL59, RDLJ-DCBA, DCBA-DDAA, DMDD-DMDE, D9CT-HD11	LADO-HHC, RIVA-PHYSO, DOP-HD11, DOP-R6E, HUX-CNBA, HUW-HHC, HUW-RDL59, CNBRPL-TCNT, CNBRPL-HD11, GLDC-CNBD, GLDC-RDLA, GLDC-DCBA, OMCNB-RDLJ, PRT-RDLA, THC-THCP, THC-D9CT, THC-HD11, THC-R6E, THCP-D9CT, THCP-HD11, CNBD-DCBA, CNBD-DDAA, ARAC1-RDLI, ARAC2-MCDD, ARAC2-RDLK, MCDC-DMDD, MCDC-DMDE, RDLA-DCBA, RDLA-DDAA, RDLH-RDL59, RDLH-DMDE, DCBA-DDAA, DMDD-DMDE, D9CT-HD11, D9CT-R6E, HD11-R6E	GNT-PHYSO, RIVA-PHYSO, CNBRPL-TCNT, CNBRPL-HD11, PRT-MCRA, THC-THCP, THC-D9CT, THC-HD11, THCP-D9CT, ARAC2-RDLK, RDLA-DCBA, RDLA-DDAA, RDLH-RDL59, DCBA-DDAA, DMDD-DMDE, D9CT-HD11

**Table 2:** PC loadings for remaining descriptors after loading analysis. Colors were used to help visualize the scale of the loadings.

	PC1	PC2	PC3
MW	0.60	0.06	−0.39
Num. Aromatic Rings	0.48	−0.21	0.85
BBB	0.33	0.88	0.05
Score (kcal/mol)	−0.55	0.42	0.35

## Data Availability

The source code (MolSimEx, Molecular Similarity Explorer) of this study is freely available at GitHub (https://github.com/Rafael-Campos-unb/MolSimEx) (accessed on 3 November 2025).

## Supplementary Materials

The following supporting information can be downloaded at: https://www.mdpi.com/article/10.3390/ijms1010000/s1. References [59,100,101,102,103,104,105,106,107,108,109,110,111,112] are cited in the supplementary materials.
