# Supplementary material for: Molecular Modeling and Analysis of Cannabinoid and Cannabinoid-like Molecules Combining K-Means Clustering with Pearson Correlation and PCA
Source: Int J Mol Sci. 2025 Nov 27;26(23):11520. doi: 10.3390/ijms262311520 (PMC12691981; doi:10.3390/ijms262311520)
Supplement: Supplementary file 1 [file ijms-26-11520-s001.zip › ijms-3924456-supplementary.pdf]

Molecular modeling and analysis of cannabinoid and cannabinoid-like molecules combining K-means clustering with Pearson correlation and PCA

Rafael Campos,† Érica C. M. Nascimento,‡ and João B. L. Martins*,†,‡

†*Department of Pharmacy, Faculty of Health Sciences, University of Brasília, Brasília, Brazil*

‡*Institute of Chemistry, University of Brasília, Brasília, Brazil*

E-mail: lopes@unb.br

Phone: +556131073886

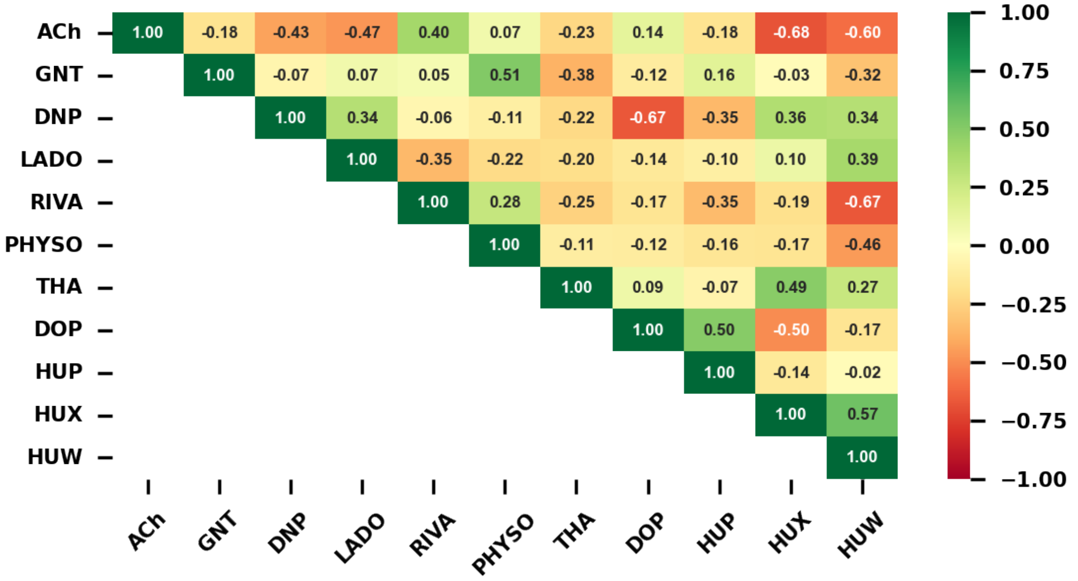

Figure S1: Validation of Similarity Matrix using classicals AChE inhibitors (AChEIs) with ADMET + EE data.

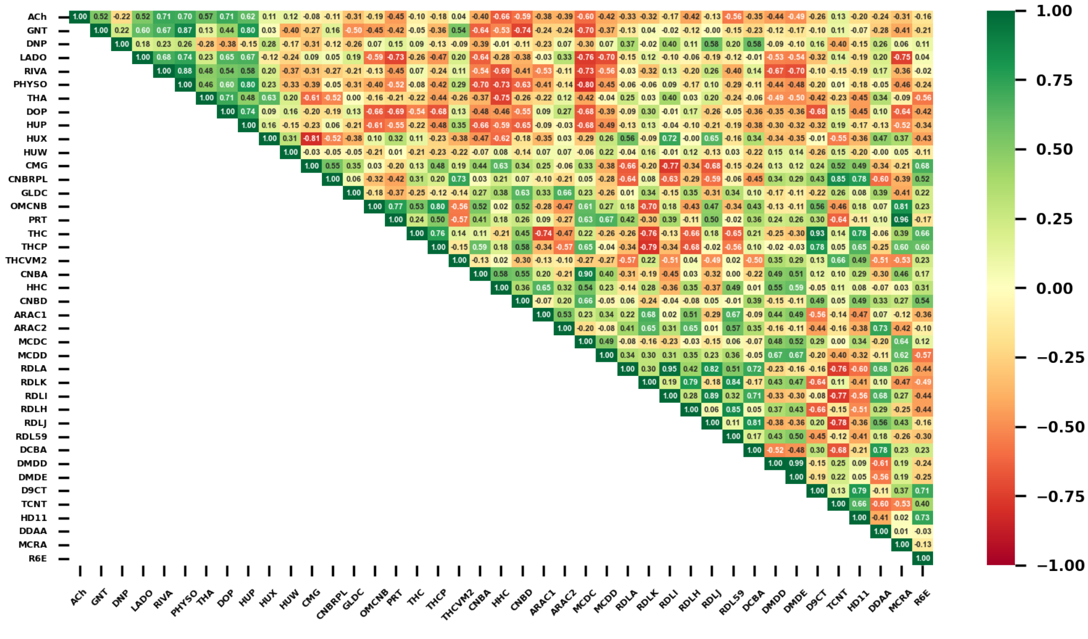

Figure S2: Analysis of Similarity between molecules studied with classicals AChE inhibitors (AChEIs) using the ADMET data of canabinnoids. The known AChEIs were also included.

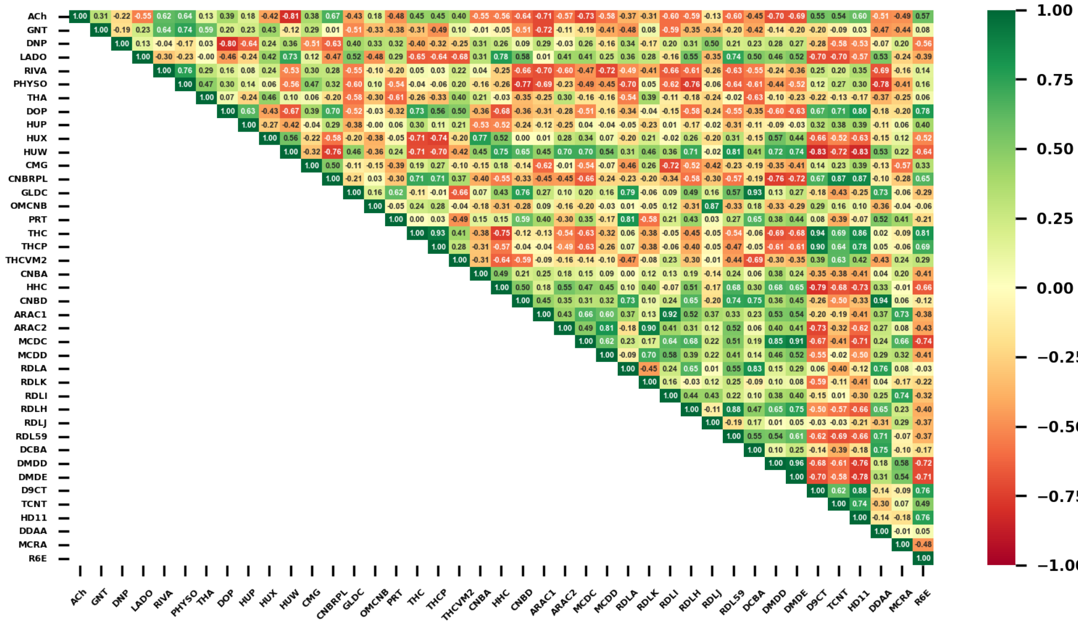

Figure S3: Analysis of Similarity between molecules studied with classicals AChE inhibitors (AChEIs) using the electronic structure data of canabinnoids. The known AChEIs were also included.

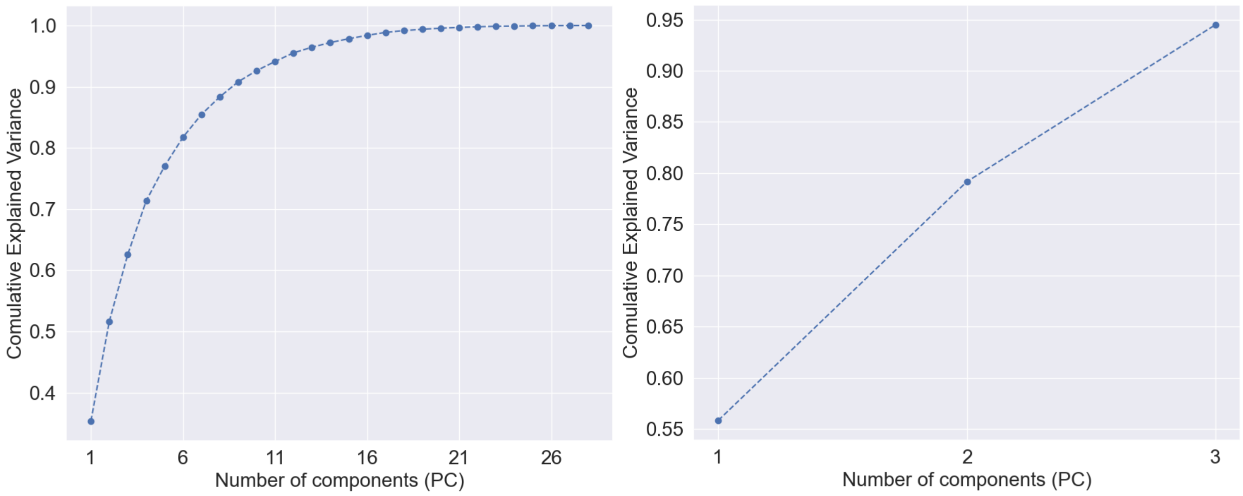

Figure S4: Explained variance based on ADMET and ES data for principal components before (left) and after (right) dimensionality reduction. In the PCA approach, the value of k was assumed to be equal to the number of principal components (PCs) that collectively contributed to almost all of the data variance.

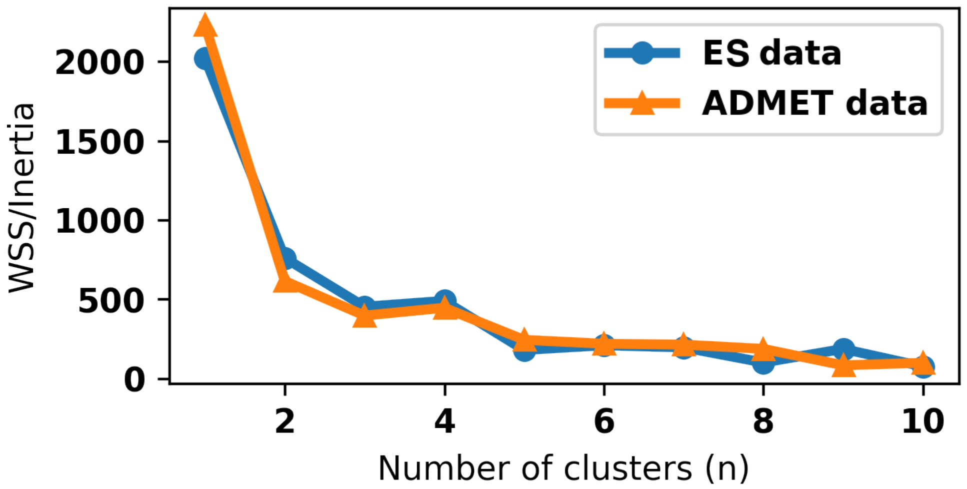

Figure S5: WSS values depending on the number of clusters for the ADMET and ES data. The value of k obtained by the elbow method was k = 5 for ADMET and ES data, with the maximum value of the WSS being greater (> 2000) for the ADMET data than for the ES data.

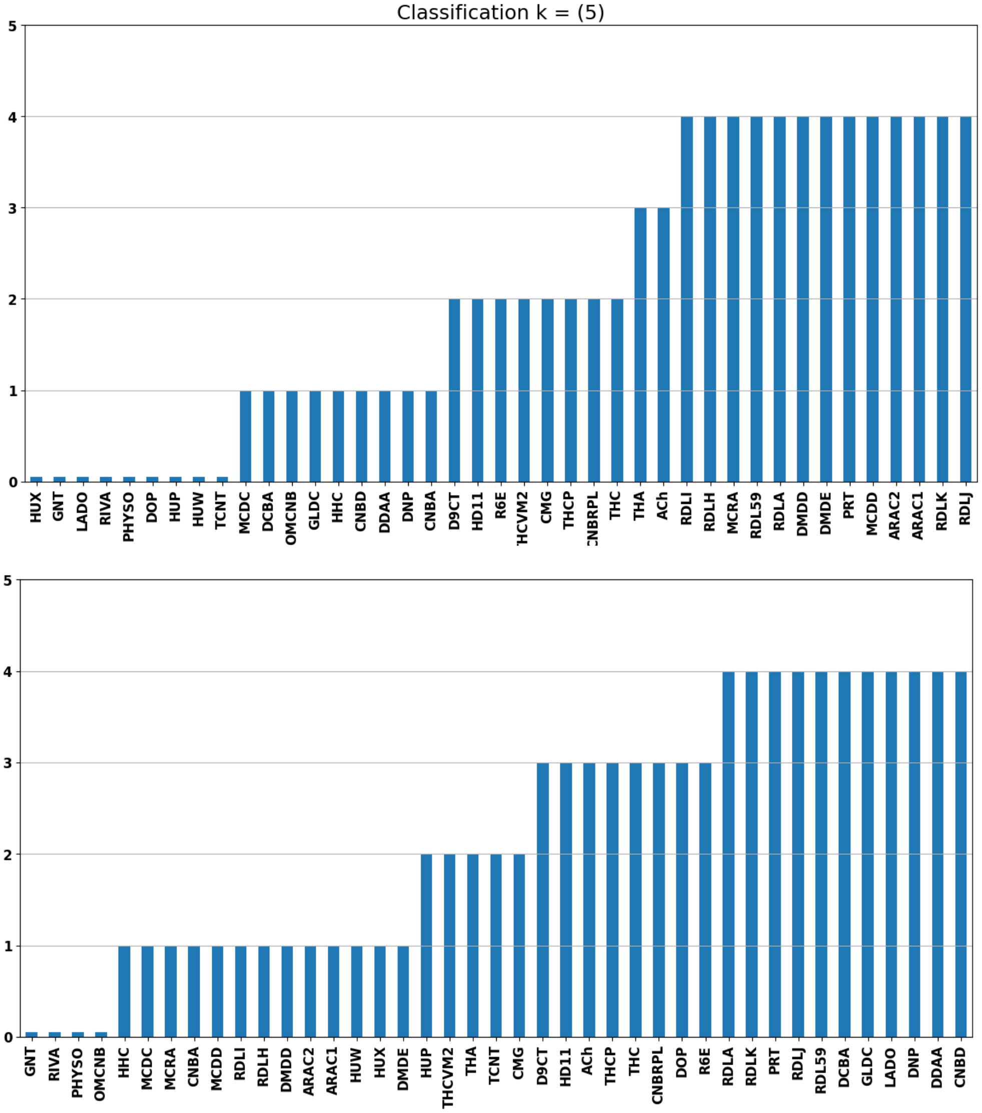

Figure S6: K-means with ADMET data (top) and ES data (bottom). The results of applying the K-means algorithm, which uses a correlation matrix, $\mathbf{M_{corr}}$, as the data space, to the ADMET and ES data separately, with k equal to 5.

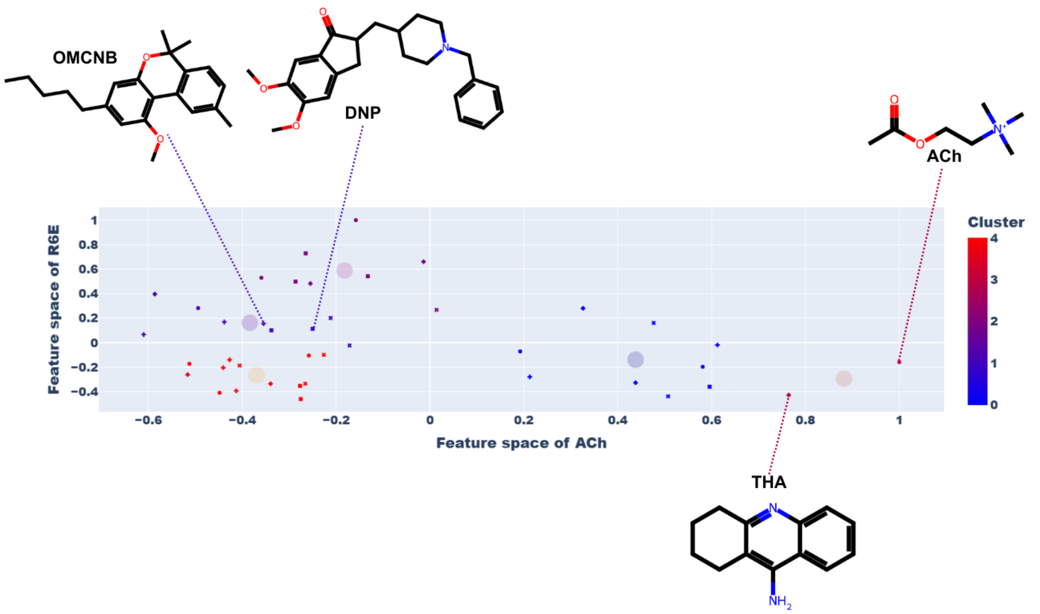

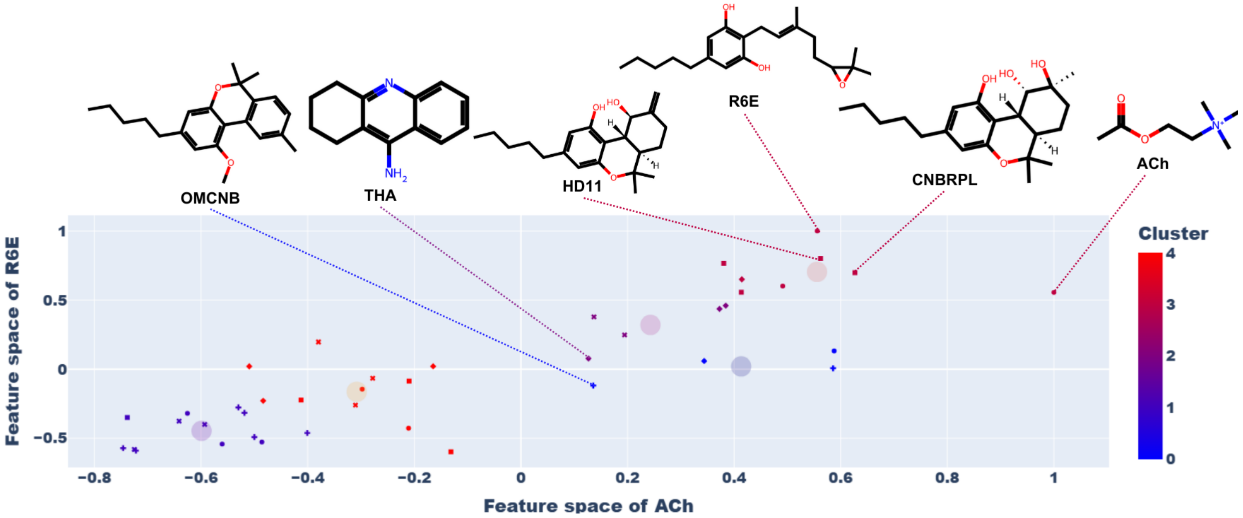

Figure S7: Comparative analysis between the ADMET and ES data. K-means with ADMET data (top) and ES data (bottom) with k=5, considering ACh and classical AChEIs. ACh is a separated group.

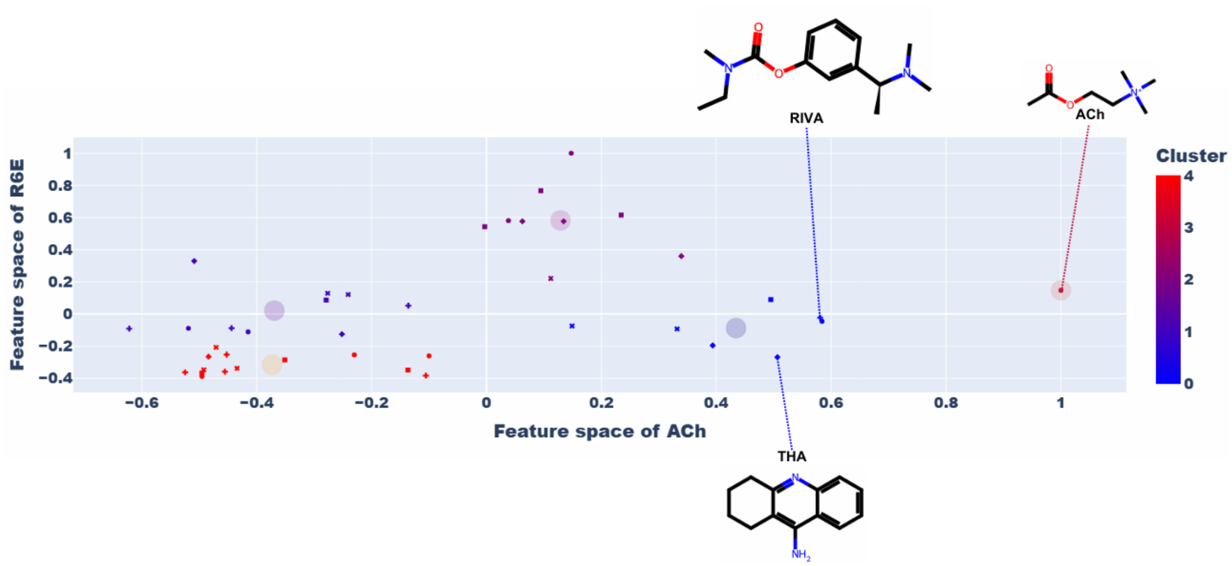

Figure S8: Rearrangement of clusters using both ADMET and EE data with k=5. ACh is in a separated group.

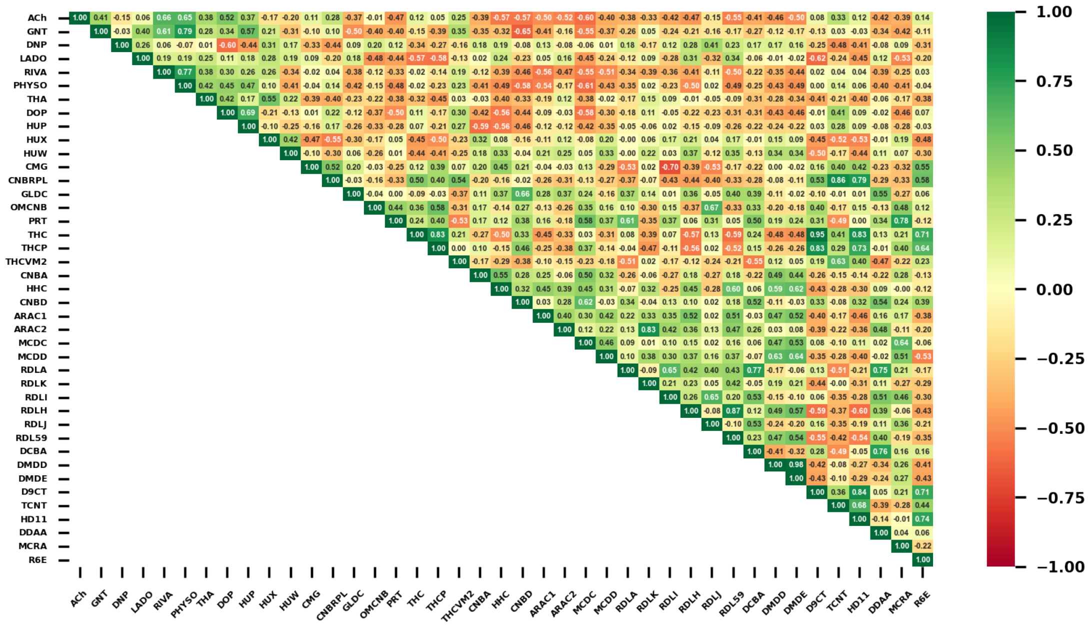

Figure S9: Analysis of Similarity between molecules studied with classicals AChE inhibitors (AChEIs) using at the same time ADMET+EE data.

	PC1	PC2	PC3	PC4	PC5
**HOMO-4 (eV)**	0.28	-0.02	0.00	-0.07	0.10
**HOMO-3 (eV)**	0.28	-0.02	0.03	-0.08	0.10
**HOMO-2 (eV)**	0.26	0.10	-0.01	-0.23	0.14
**HOMO-1 (eV)**	0.23	0.18	0.01	-0.26	0.07
**HOMO (eV)**	0.20	0.12	-0.00	-0.24	0.18
**LUMO (eV)**	-0.09	0.34	-0.05	-0.04	0.15
**LUMO+1 (eV)**	-0.14	0.35	0.05	-0.02	-0.07
**LUMO+2 (eV)**	-0.14	0.35	0.04	-0.09	0.02
**LUMO+3 (eV)**	-0.17	0.36	-0.01	-0.02	-0.01
**LUMO+4 (eV)**	-0.19	0.32	0.03	-0.07	0.04
**H1 (a.u.)**	0.16	0.10	0.26	-0.23	-0.03
**CHARGE HETEROA**	0.08	-0.08	-0.31	0.36	0.10
**H1-H2 (A)**	0.10	-0.02	0.29	0.04	0.09
**Molecular size (A)**	0.25	0.18	-0.06	0.16	-0.05
**VOLUME (cm^3)**	0.00	-0.01	0.11	0.36	0.35
**MW**	0.26	0.09	0.03	0.28	-0.06
**Num Aromatic Rings**	0.22	-0.17	-0.12	-0.03	-0.16
**Fraction Csp3**	-0.15	0.25	-0.02	0.29	-0.10
**Rotatable bonds**	0.04	0.18	-0.01	0.23	0.09
**H-bond acceptors**	0.11	0.08	0.39	0.25	0.05
**H-bond donors**	0.14	0.04	0.43	-0.06	-0.16
**MR**	0.27	0.10	-0.06	0.25	-0.02
**TPSA**	0.12	0.00	0.47	0.10	-0.15
**Consensus Log P**	0.24	0.16	-0.24	-0.02	0.02
**ESOL Log S**	-0.27	-0.12	0.19	-0.05	0.01
**BBB permeant**	0.07	0.12	0.03	0.28	-0.36
**Drugscore**	-0.05	0.05	0.08	0.09	0.67
**Druglikeness**	-0.06	-0.28	0.16	0.06	0.26
**Score(kcal/mol)**	-0.26	-0.11	0.13	0.04	-0.09

Figure S10: PCA loadings calculated for all descriptors (ADMET and EE).

Table S1: Virtual Screening using Autodock Vina (Part 1)

Molecule	SMILES	CID/C_id	Score/Vina (kcal/mol)
gama-Eudesmyl-D9-trans-Tetrahydrocannabinolate	CCCCCc1cc2OC(C)(C)[C@H]3[C@H](c2c(c1C(=O)OC([C@@H]1CC[C@@]2(C(=C(C)CCC2)C1)C)(C)C)O)C=C(CC3)C	24862526	-9.9
alfa-Terpinyl-D9-trans-Tetrahydrocannabinolate	CCCCCc1cc2OC(C)(C)[C@H]3[C@H](c2c(c1C(=O)OC([C@@H]1CCC(=CC1)C)(C)C)O)C=C(CC3)C	-	-7.9
alfa-Cadinyl-D9-trans-Tetrahydrocannabinolate	CCCCCc1cc2OC(C)(C)[C@H]3[C@H](c2c(c1C(=O)O[C@@]1(C)CC[C@H]([C@@H]2[C@H]1CCC(=C2)C)C(C)C)O)C=C(CC3)C	-	-9.1
VCE-004-8	CCCCCC1=C(NCc2ccccc2)C(=O)C(=C(C1=O)O)[C@H]1C=C(C)CC[C@H]1C(=C)C	-	-8.3
VCE-003	CCCCCC1=C(C/C=C(/CCC=C(C)C)\C)C(=O)C=C(C1=O)O	44139743	-8.9
Trans-Cannabitriol	CCCCCc1cc(O)c2c(c1)OC(C1=C2[C@H](O)[C@@H](O)CC1)(C)C	155804790	-10
Trans-arachidin-2	CC(=CCc1c(O)cc(cc1O)CCc1ccc(cc1)O)C	92012758	-11.2
Trans-arachidin-1	CC(/C=C/c1c(O)cc(cc1O)/C=C/c1ccc(c(c1)O)O)C	11220670	-13
THC-O-Acetate	CCCCCc1cc(OC(=O)C)c2c(c1)OC([C@H]1[C@H]2C=C(C)CC1)(C)C	198013	-9.9
Tetrahydrocannabiphorol	CCCCCCCc1cc(O)c2c(c1)OC([C@H]1[C@H]2C=C(C)CC1)(C)C	6453074	-11.6
Tetrahydrocannabinol-epoxide	CCCCCc1cc(O)c2c(c1)OC([C@@H]1[C@@H]2[C@@H]2O[C@]2(CC1)C)(C)C	-	-10.6
TetrahidrocanabivarinaM3	C[C@H](Cc1cc(O)c2c(c1)OC([C@H]1[C@H]2C=C(CC1)C(=O)O)(C)C)O	-	-10.5
Tetrahydrocannabivarine-M2	CCCc1cc(O)c2c(c1)OC([C@H]1[C@H]2C=C(CC1)C(=O)O)(C)C	101123021	-11.2
TetrahidrocanabivarinaM1	CCCc1cc(O)c2c(c1)OC([C@H]1[C@H]2C=C(CO)CC1)(C)C	137518224	-11.2
Tetrahidrocanabivarina	CCCc1cc(O)c2c(c1)OC([C@H]1[C@H]2C=C(C)CC1)(C)C	93147	-10.6
Sesquicannabigerol	CCCCCc1cc(O)c(c(c1)O)C/C=C(/CC/C=C(/CCC=C(C)C)\C)\C	54669855	-8.3
Rhododaurichromanic-acid-A	CC(=CCCc1cc2O[C@@]3(C)CC[C@@H]4[C@H]3[C@H](c2c(c1C(=O)O)O)C4(C)C)C	-	-9.7
Radulanin L	CC1=CCc2c(OC1)cc(cc2O)/C=C/c1ccccc1O	-	-12.1
Radulanin K	OC(=O)c1c(/C=C/c2ccccc2)cc2c(c1O)[C@H]1C[C@]1(CO2)C	C00015861	-12.1
Radulanin J	COc1cc(/C=C/c2ccccc2)cc2c1[C@H]1C[C@]1(CO2)C	14804135	-12.3
Radulanin I	Oc1cc(/C=C/c2ccccc2)cc2c1[C@H]1C[C@]1(CO2)C	14804134	-12.3
Radulanin H	CC1=CCc2c(OC1)cc(c(c2O)C(=O)O)/C=C/c1ccccc1	C00015858	-11.1
Radulanin A	CC1=CCC2=C(C=C(C=C2OC1)CCC3=CC=CC=C3)O	9970905	-12.9
Radulanin 59	CC1=CCc2c(OC1)c(C(=O)O)c(cc2O)/C=C/c1ccccc1	-	-13.3
rac-6-epoxycannabigerolic-acid	CCCCCc1cc(O)c(c(c1C(=O)O)O)C[C@H]1O[C@]1(C)CCC=C(C)C	-	-9
rac-6-epoxycannabigerol	CCCCCc1cc(O)c(c(c1)O)C[C@H]1O[C@]1(C)CCC=C(C)C	168310608	-11.3
Perrottetinen-acid	CC1=C[C@H]2[C@@H](CC1)C(C)(C)Oc1c2c(O)c(c(c1)/C=C/c1ccccc1)C(=O)O	-	-12.8
Perrottetinen	CC1=C[C@H]2[C@@H](CC1)C(C)(C)Oc1c2c(O)cc(c1)/C=C/c1ccccc1	24766094	-12.4
Oxo-Cannabitriol	CCCCCc1cc(O)c2c(c1)OC(C1=C2C(=O)[C@](CC1)(C)O)(C)C	-	-9.9
O-Propyl-D9-trans-tetrahydrocannabinol	CCCOc1cc(CCCCC)cc2c1[C@@H]1C=C(C)CC[C@H]1C(O2)(C)C	-	-8.6
O-propylcannabidiol	CCCOc1cc(CCCCC)cc(c1[C@H]1C=C(C)CC[C@@H]1C(=C)C)O	-	-9.7
O-prophylCannabinol	CCCOc1cc(CCCCC)cc2c1c1cc(C)ccc1C(O2)(C)C	-	-10.4
O-Pentyl-D9-trans-tetrahydrocannabinol	CCCCCOc1cc(CCCCC)cc2c1[C@@H]1C=C(C)CC[C@H]1C(O2)(C)C	164990027	-8.2
O-pentylcannabidiol	CCCCCOc1cc(CCCCC)cc(c1[C@H]1C=C(C)CC[C@@H]1C(=C)C)O	-	-9.2
O-PenthylCannabinol	CCCCCOc1cc(CCCCC)cc2c1c1cc(C)ccc1C(O2)(C)C	-	-9.6
O-metthylCannabinol	CCCCCc1cc(OC)c2c(c1)OC(c1c2cc(C)cc1)(C)C	628150	-10.1
nor-Cannabivarina	CCc1cc(O)c2c(c1)OC(c1c2cc(C)cc1)(C)C	59444399	-10.1
nor-Cannabitriol	CCCc1cc(O)c2c(c1)OC(C1=C2[C@@H](O)[C@](CC1)(C)O)(C)C	59444417	-10.9
nor-Cannabinol	CCCCc1cc(O)c2c(c1)OC(c1c2cc(C)cc1)(C)C	59444392	-11.5
N-araquidonoilglicierol	CCCCC/C=C\C/C=C\C/C=C\C/C=C\CCCC(=O)NCC(=O)O	5283389	-7.5
methylen-bis-D9-trans-tetrahydrocannabinol	CCCCCc1cc2OC(C)(C)[C@H]3[C@H](c2c(c1Cc1c(CCCCC)cc2c(c1O)[C@@H]1C=C(C)CC[C@H]1C(O2)(C)C)O)C=C(CC3)C	102487751	-7.6
Machaeridiol D	O[C@H]1C[C@H]2[C@H](C[C@H]1C)c1c(O)cc(cc1OC2(C)C)c1cc2c(o1)cccc2	10861764	-11.2
Machaeridiol C	CC1CCC(C(C1)C2=C(C=C(C=C2O)C3=CC4=CC=CC=C4O3)O)C(=C)C	10882982	-12.6
Machaeriol B	C[C@H]1CC[C@H]2[C@H](C1)c1c(O)cc(cc1OC2(C)C)c1cc2c(o1)cccc2	10384052	-11.8
Machaeriol A	C[C@H]1CC[C@H]2[C@H](C1)c1c(O)cc(cc1OC2(C)C)/C=C/c1ccccc1	162870568	-11.3
Machaeriol C	C[C@@H]1CC[C@H]([C@@H](C1)c1c(O)cc(cc1O)c1cc2c(o1)cccc2)C(=C)C	-	-10.9
Machaeriol B	C[C@@H]1CC[C@H]([C@@H](C1)c1c(O)cc(cc1O)/C=C/c1ccccc1O)C(=C)C	-	-11.8
Machaeridiol A	C[C@@H]1CC[C@H]([C@@H](C1)c1c(O)cc(cc1O)/C=C/c1ccccc1)C(=C)C	-	-9.6
Linderatin	CC1=C[C@H]([C@@H](CC1)C(=C)C)c1c(O)cc(c(c1O)C(=O)Cc1ccccc1)O	-	-8.6
Isotetrahydrocannabivarina	CCCc1cc(O)c2c(c1)O[C]1(=CC2=C(C=C1)C(=C)C)C	-	-10.8
Isotetrahydrocannabinol	CCCCCc1cc(O)c2c(c1)O[C]1(=CC2=C(C=C1)C(=C)C)C	-	-12.1
Iso-Cannabitriol	CCCCCc1cc(O)c2c(c1)OC(C1=C2[C@@H](O)[C@]([C@@H](C1)O)(C)O)(C)C	-	-10.8
hydroxy-D9-11-hexahydrocannabinol	CCCCCc1cc(O)c2c(c1)OC([C@H]1[C@H]2[C@@H](O)C(=C)CC1)(C)C	122180086	-10.9
Hydroxy-helicannabigerol	C/C(=C\Cc1c(O)cc(c(c1O)C(=O)O)CCc1ccc(cc1)O)/CCC=C(C)C	-	-11.8
HU-313	CCCCCC1=CC(=O)C(=C(C1=O)O)[C@H]1C=C(C)CC[C@H]1C(=C)C	98454947	-10
Hexahydrocannabinol	CCCCCc1cc(O)c2c(c1)OC([C@H]1[C@H]2C[C@H](C)CC1)(C)C	11067065	-11.8
Heli-cannabigerol	C/C(=C\Cc1c(O)cc(cc1O)CCc1ccccc1)/CCC=C(C)C	14060723	-12
Glepidotin-C-51	OC(=O)c1c(/C=C/c2ccccc2)cc(c(c1O)CCC(O)(C)C)O	-	-12.7
Glepidotin-C	O[C@H](C(=C)C)Cc1c(O)cc(cc1O)CCc1ccccc1	442703	-11.5
gama-Eudesmyl-cannabigerolate	CCCCCc1cc(O)c(c(c1C(=O)OC([C@@H]1CC[C@]2(C(=C(C)CCC2)C1)C)(C)C)O)C/C=C(/CCC=C(C)C)\C	-	-9.9

Table S1: Virtual Screening using Autodock Vina (Part 2)

Molecule	SMILES	CID/C_id	Score/Vina (kcal/mol)
gama-Cadinyl-cannabigerolate	CCCCCc1cc(O)c(c(c1C(=O)O[C@@]1(C)CC[C@@H]([C@@H]2[C@@H]1CCC(=C2)C)C(C)C)O)C/C=C(/CCC=C(C)C)\C	-	-8.2
Ferruginene-C	CC(=C)[C@H](CCC(=C)[C@@H]1CCC(=C[C@H]1c1c(O)cc(cc1O)C)C)O	101796996	-9.7
Ferruginen-B	Cc1cc(O)c2c(c1)O[C@@H]1[C@H]2[C@H](CC[C@@]1(C)O)C(=C)C/C=C/C(O)(C)C	-	-8.6
Ferruginen-A	Cc1cc(O)c2c(c1)O[C@@H]1[C@H]2[C@H](CC[C@@]1(C)O)C(=C)CC[C@@H](C(=C)C)O	-	-9.6
Ethyl-Cannabitriol	CCCCCc1cc(O)c2c(c1)OC(C1=C2[C@@H](OCC)[C@](CC1)(C)O)(C)C	59444384	-8.9
Dronabinol	CCCCCc1cc(O)c2c(c1)OC([C@H]1[C@H]2C=C(C)CC1)(C)C	16078	-11.6
Desmodianone-A	CC1=C[C@H]2[C@@H](CC1)C(C)(C)Oc1c2c(O)cc(c1)[C@H]1COc2c(C1=O)c(O)c(c(c2)O)C	-	-9.5
Desmodianone-E	CC1=CC2C(CC1)C(C)(C)Oc1c2cc(c(c1)O)C1COc2c(C1=O)c(O)c(c(c2)O)C	10993817	-11.9
Desmodianone-D	Oc1cc(cc2c1[C@@H]1[C@H]3[C@@](O2)(C)CC[C@@H]3C1(C)C)[C@H]1COc2c(C1=O)c(O)c(c(c2)O)C	11102162	-11.7
Desmodianone-C	C/C(=C\Cc1c(O)cc(cc1O)[C@H]1COc2c(C1=O)c(O)c(c(c2)O)C)/CCC=C(C)C	-	-9.4
Deprenyl-O-methyl-cannabigerolic-acid	CCCCCc1cc(OC)c(c(c1C(=O)O)O)CC=C(C)C	23874497	-9.8
Demethyl-decacarbo-Amorfrutin-A	CC(=CCc1c(O)cc(cc1O)CCc1ccccc1)C	442715	-11.1
DemethylAmorfrutin-A	CC(=CCc1c(O)cc(c(c1O)C(=O)O)CCc1ccccc1)C	9945203	-11.6
Delta-9-tetrahydrocannabivarinic-acid	CCCc1cc2OC(C)(C)[C@H]3[C@H](c2c(c1C(=O)O)O)C=C(CC3)C	59444416	-9.5
Delta-9-tetrahydrocannabiorcol	CC1=C[C@@H]2[C@@H](CC1)C(C)(C)Oc1c2c(O)cc(c1)C	22805649	-9.7
delta-9-tetrahydrocannabinol-C4	CCCCc1cc(O)c2c(c1)OC([C@H]1[C@H]2C=C(C)CC1)(C)C	6453891	-11.2
Delta-9-cis-tetrahydrocannabinol	CCCCCc1cc(O)c2c(c1)OC([C@@H]1[C@H]2C=C(C)CC1)(C)C	12831993	-11.9
delta-8-Tetrahydrocannabinol-acid	CCCCCc1cc2OC(C)(C)[C@@H]3[C@@H](c2c(c1C(=O)O)O)CC(=CC3)C	-	-9.6
delta-8-Tetrahydrocannabinol	CCCCCc1cc(O)c2c(c1)OC([C@H]1[C@@H]2CC(=CC1)C)(C)C	86308458	-10.9
delta1-Tetrahydrocannabiorcol	CC1=C[C@@H]2[C@@H](CC1)C(C)(C)Oc1c2c(O)cc(c1)C	22805649	-9.5
dehydrocannabifuran	CCCCCC1=CC2OC3C(C2=C(C1)O)C(CCC3C)C(=C)C	-	-11.8
Decarboxyamorfrutin-B	COc1cc(CCc2ccccc2)c(c(c1C/C=C(/CCC=C(C)C)\C)O)C(=O)O	24739090	-9.5
DecarboxyAmorfrutin-A	COc1cc(CCc2ccccc2)cc(c1CC=C(C)C)O	14805915	-12.6
Daurichromenic-acid	C/C(=C\CC[C@]1(C)C=Cc2c(O1)cc(c(c2O)C(=O)O)C)/CCC=C(C)C	11696381	-11.5
D9-trans-Tetrahydrocannabiorcolic acid	CC1=C[C@@H]2[C@@H](CC1)C(C)(C)Oc1c2c(O)c(c(c1)C)C(=O)O	59444386	-10.3
D9-trans-Tetrahydrocannabinolic-acid-B	CCCCCc1cc(O)c2c(c1C(=O)O)OC([C@H]1[C@H]2C=C(C)CC1)(C)C	46889976	-11.1
D9-trans-Tetrahydrocannabinolic-acid-A	CCCCCc1cc2OC(C)(C)[C@H]3[C@H](c2c(c1C(=O)O)O)C=C(CC3)C	98523	-10.2
D9-trans-nor-Tetrahydrocannabinolic acid	CCCCc1cc2OC(C)(C)[C@H]3[C@H](c2c(c1C(=O)O)O)C=C(CC3)C	59444388	-10
D9-Tetrahydrocannabinolic-acid	CCCCCc1cc2OC(C)(C)[C@H]3[C@H](c2c(c1C(=O)O)O)C=C(CC3)C	98523	-10.4
Confluentin	CC(=CCC[C@]1(C)C=Cc2c(O1)c(C)c(c(c2)O)C(=O)O)C	-	-10.7
Citrylidenecannabis	CCCCCc1cc2O[C@]3(C)CC[C@H]4[C@H](c2c(c1)OC4(C)C)C3	101820920	-9.5
Chricanine-B	Oc1cc(/C=C/c2ccccc2)cc2c1C[C@@H](O)C(O2)(C)C	163193809	-12
Chlorcannabiorcichromenic-acid	CC(=CCC[C@@]1(C)C=Cc2c(O1)c(Cl)c(c(c2O)C(=O)O)C)C	-	-10.8
Chiricanin-A	CC(=CCc1c(O)cc(cc1O)CCc1ccccc1)C	442715	-11.6
THC	CCCCCC1=CC(=C2C3C=C(CCC3C(OC2=C1)(C)C)C)O	2978	-11.4
CBDA-THC-ester	CCCCCc1cc(O)c2c(c1)OCC1=C2[C@H](O)[C@@](CC1)(C)OOC(=O)c1c(CCCCC)cc(c(c1O)[C@H]1C=C(C)CC[C@@H]1C(=C)C)O	-	-9.6
Carmagerol	CCCCCc1cc(O)c(c(c1)O)C/C=C(\CC[C@@H](C(O)(C)C)O)/C	44586785	-12
Cannflavin-A	COc1cc(ccc1O)c1cc(=O)c2c(o1)cc(c(c2O)CC/C=C(/CCC=C(C)C)\C)O	-	-10.8
Cannabivarina	CCCc1cc(O)c2c(c1)OC(c1c2cc(C)cc1)(C)C	622545	-9.9
Cannabitriol	CCCCCc1cc(O)c2c(c1)OC(C1=C2[C@H](O)[C@@](CC1)(C)O)(C)C	156460	-9.6
Cannabistilbene-I	COc1cc(CCc2ccc(c(c2)CC=C(C)C)O)cc(c1)O	146349	-7.9
Cannabispiran	COc1cc(O)c2c(c1)CCC12CCC(=O)CC1	162936	-9.5
Cannabispiradienone	COc1cc(O)c2c(c1)CC[C@@]12C=CC(=O)C=C1	90475437	-10
Cannabisin-G	COc1cc(ccc1O)/C=C(\C(=C/c1ccc(c(c1)OC)O)\C(=O)NCCc1ccc(cc1)O)/C(=O)NCCc1ccc(cc1)O	10438919	-11.4
Cannabisin-A	Oc1ccc(cc1)CCNC(=O)c1cc2cc(O)c(cc2c(c1C(=O)NCCc1ccc(cc1)O)c1ccc(c(c1)O)O)O	15086398	-12.6
Cannabiscitrin	OC[C@H]1O[C@@H](Oc2cc(cc(c2O)O)C2OC3C=C(O)CC(=C3C(=O)C2O)O)[C@@H]([C@H]([C@@H]1O)O)O	-	-11.3
Cannabisativine	CCCCC[C@H]([C@H]([C@@H]1C=CC[C@@H]2N1CCCNCCCCNC(=O)C2)O)O	442846	-10.1
Cannabiripsol	CCCCCc1cc(O)c2c(c1)OC([C@H]1[C@H]2[C@H](O)[C@@](CC1)(C)O)(C)C	192007	-12.5
Cannabioxepane	CCCCCC1=CC2OC3C4C2=C(C1)OCC(=C)C4CCC3C	-	-11.1
Cannabiorcol	Cc1ccc2c(c1)c1c(O)cc(cc1OC2(C)C)C	59444404	-10.7
Cannabiorcicitran	Cc1cc2O[C]3(=CC4=C(C(Oc(c1)c24)(C)C)C=C3)C	-	-10
Cannabiorcichromenic-acid	CC(=CCC[C@@]1(C)C=Cc2c(O1)cc(c(c2O)C(=O)O)C)C	101737129	-10.4
Cannabiorcichromene	CC(=CCC[C@]1(C)C=Cc2c(O1)cc(cc2O)C)C	162881404	-9.9
Cannabinolic-acid	CCCCCc1cc2OC(C)(C)c3c(c2c(c1C(=O)O)O)cc(cc3)C	3081990	-10.6
cannabinol-C4	CCCCc1cc(O)c2c(c1)OC(c1c2cc(C)cc1)(C)C	59444392	-10.3
cannabinol-C2	CCc1cc(O)c2c(c1)OC(c1c2cc(C)cc1)(C)C	59444399	-10.2
O-methyl-Cannabinol	CCCCCc1cc(OC)c2c(c1)OC(c1c2cc(C)cc1)(C)C	628150	-12.5
Cannabinodivarin	CCCc1cc(O)c(c(c1)O)c1cc(C)ccc1C(=C)C	59444390	-10.3

Table S1: Virtual Screening using Autodock Vina (Part 3)

Molecule	SMILES	CID/C_id	Score/Vina (kcal/mol)
Cannabinodiol	CCCCCc1cc(O)c(c(c1)O)c1cc(C)ccc1C(=C)C	11551346	-11.2
Cannabinerolic-acid	CCCCCc1cc(O)c(c(c1C(=O)O)O)C/C=C(\CCC=C(C)C)/C	9998639	-9.7
Cannabimovone	CCCCCc1cc(O)c(c(c1)O)[C@H]1[C@@H](C[C@H]([C@@H]1O)C(=O)C)C(=C)C	46217279	-11
Cannabiglendol	CCCc1cc(O)c2c(c1)O[C@@]1(C[C@H]2[C@H](CC1)C(O)(C)C)C	-	-9.5
Cannabigerovarinic-acid	CCCc1cc(O)c(c(c1C(=O)O)O)C/C=C(/CCC=C(C)C)\C	59444383	-9.7
Cannabigerovarin	CCCc1cc(O)c(c(c1)O)C/C=C(/CCC=C(C)C)\C	59444407	-10.2
Cannabigeroquinone	CCCCCC1=CC(=O)C(=CC1=O)C/C=C(/CCC=C(C)C)\C	-	-10.3
Cannabigerolic-acid-monomethyl-ether	CCCCCc1cc(OC)c(c(c1C(=O)O)O)C/C=C(/CCC=C(C)C)\C	24739091	-9.3
Cannabigerolic-acid	CCCCCc1cc(O)c(c(c1C(=O)O)O)C/C=C(/CCC=C(C)C)\C	6449999	-9.5
Cannabigerol-monomethyl-ether	CCCCCc1cc(O)c(c(c1)OC)C/C=C(/CCC=C(C)C)\C	13864080	-7.9
Cannabigerol	CCCCCc1cc(O)c(c(c1)O)C/C=C(/CCC=C(C)C)\C	5315659	-9.9
Cannabifuran	CCCCCC1CC(O)C2C(C1)OC1C2=C(C=C[C@@H]1C)C(C)C	-	-9.9
Cannabielsoin	CCCCCc1cc(O)c2c(c1)O[C@H]1[C@@H]2[C@@H](CC[C@]1(C)O)C(=C)C	162113	-8.7
Cannabielsoic-acid-B	CCCCCc1cc(O)c2c(c1C(=O)O)O[C@@H]1[C@H]2[C@H](CC[C@@]1(C)O)C(=C)C	-	-9.1
cannabielsoic-acid-A	CCCCCc1cc2O[C@H]3[C@@H](c2c(c1C(=O)O)O)[C@@H](CC[C@]3(C)O)C(=C)C	59444405	-9.6
Cannabidivarinic-Acid	CCCc1cc(O)c(c(c1C(=O)O)O)[C@@H]1C=C(C)CC[C@H]1C(=C)C	59444387	-10.3
Cannabidivarina	CCCc1cc(O)c(c(c1)O)[C@@H]1C=C(C)CC[C@H]1C(=C)C	11601669	-9.8
Cannabidiorcol	CC1=C[C@@H]([C@H](CC1)C(=C)C)c1c(O)cc(cc1O)C	124350795	-9.4
cannabidiolate	CCCCCc1cc(O)c(c(c1C(=O)O)O)[C@@H]1C=C(C)CC[C@H]1C(=C)C	160570	-11.7
Cannabidiol-3-monomethyl-ether	CCCCCc1cc(O)c(c(c1)OC)[C@@H]1C=C(C)CC[C@H]1C(=C)C	164905	-9.3
Cannabidiol	CCCCCc1cc(O)c(c(c1)O)[C@@H]1C=C(C)CC[C@H]1C(=C)C	644019	-11.2
Cannabidibutol	CCCCc1cc(O)c(c(c1)O)[C@@H]1C=C(C)CC[C@H]1C(=C)C	59444413	-10.5
Cannabicyclovarin	CCCc1cc2O[C@]3(C)CC[C@H]4[C@@H]3[C@@H](c2c(c1)O)C4(C)C	59444397	-9.3
Cannabicyclolic-Acid	CCCCCc1cc2O[C@]3(C)CC[C@H]4[C@@H]3[C@@H](c2c(c1C(=O)O)O)C4(C)C	59444422	-10.8
Cannabicyclol	CCCCCc1cc(O)c2c(c1)O[C@]1([C@H]3[C@@H]2C([C@H]3CC1)(C)C)C	59444380	-11.2
Cannabicyclohexanol	CCCCCCCC(c1ccc(c(c1)O)[C@@H]1CCC[C@@H](C1)O)(C)C	12788231	-10.1
Cannabicoumarononic-acid	CCCCCc1cc2occ3c2c(c1C(=O)O)OC([C@H]3CCC(=O)C)(C)C	-	-12
Cannabicoumaronone-1	CCCCCc1cc2OC(C)(C)[C@H](c3c2c(c1)oc3)CCC(=O)C	-	-9.5
Cannabicoumaronone	CCCCCC1=CC2=C3C(C1)OCC3[C@@H](C(O2)(C)C)CCC(=O)C	-	-10.2
Cannabicitran	CCCCCc1cc2O[C@@]3(C)CC[C@@H]4[C@@H](c2c(c1)OC4(C)C)C3	21668222	-10.6
Cannabicicyclolic-acid	OC(=O)c1c(C)cc2c(c1O)[C@H]1[C@H]3[C@](O2)(C)CC[C@H]3C1(C)C	101542703	-10.8
Cannabicicyclol	Cc1cc2O[C@@]3(C)CC[C@@H]4[C@H]3[C@H](c2c(c1)O)C4(C)C	21668227	-9.9
Cannabichromevarinic-Acid	CCCc1cc2O[C@@](C)(CCC=C(C)C)C=Cc2c(c1C(=O)O)O	156367564	-9.9
Cannabichromevarina	CCCc1cc2O[C@@](C)(CCC=C(C)C)C=Cc2c(c1)O	139496813	-8.6
Cannabichromenate	CCCCCc1cc2O[C@@](C)(CCC=C(C)C)C=Cc2c(c1C(=O)O)O	92448135	-9
Cannabichromanone-D	CCCCCc1cc2OC(=CCC[C@@H]3C(=O)c2c(c1)OC3(C)C)C	163078176	-10
Cannabichromanone	CCCCCc1cc(O)c2c(c1)OC([C@H](C2=O)CCC(=O)C)(C)C	162929173	-10.1
cannabichromanon	CCCCCc1cc(O)c2c(c1)OC([C@@H](C2=O)CCC(=O)C)(C)C	25105340	-9.4
Canabinol-M3	C[C@H](CCCc1cc(O)c2c(c1)OC(c1c2cc(cc1)C(=O)O)(C)C)O	-	-12.8
CanabinolM2	CCCCCc1cc(O)c2c(c1)OC(c1c2cc(cc1)C(=O)O)(C)C	629782	-13.6
CanabinolM1	CCCCCc1cc(O)c2c(c1)OC(c1c2cc(CO)cc1)(C)C	3082311	-12.3
Canabinol	CCCCCc1cc(O)c2c(c1)OC(c1c2cc(C)cc1)(C)C	2543	-11.8
CanabichromenoM1	C[C@@H](CCCc1cc2O[C@@](C)(CCC=C(C)C)C=Cc2c(c1)O)O	-	-10.2
Canabichromeno	CCCCCc1cc2O[C@@](C)(CCC=C(C)C)C=Cc2c(c1)O	21668219	-9.9
Bornyl-D9-trans-Tetrahydrocannabinolate	CCCCCc1cc2OC(C)(C)[C@H]3[C@H](c2c(c1C(=O)O[C@H]1C[C@@H]2C([C@@]1(C)CC2)(C)C)O)C=C(CC3)C	24862528	-9.3
Bisnor-Cannabichromanone	CCCc1cc(O)c2c(c1)OC([C@@H](C2=O)CCC(=O)C)(C)C	-	-10.1
Bisnor-cannabielsoin	CCCc1cc(O)c2c(c1)O[C@@H]1[C@H]2[C@H](CC[C@@]1(C)O)C(=C)C	-	-11.1
Cannabielsoin	CCCc1cc(O)c2c(c1C(=O)O)O[C@@H]1[C@H]2[C@H](CC[C@@]1(C)O)C(=C)C	-	-8.7
B-Fenchyl-D9-trans-Tetrahydrocannabinolate	CCCCCc1cc2OC(C)(C)[C@H]3[C@H](c2c(c1C(=O)O[C@@H]1[C@]2(C)CC[C@H](C1(C)C)C2)O)C=C(CC3)C	-	-8.4
Armofrutin-C	C/C(=C\Cc1c(O)cc(c(c1O)C(=O)O)CCc1ccccc1)/CCC=C(C)C	25135579	-12.1
Armofrutin-B	COc1cc(CCc2ccccc2)c(c(c1C/C=C(/CCC=C(C)C)\C)O)C(=O)O	24739090	-12.1
Armofrutin-4	C/C(=C\Cc1c(O)cc(c(c1O)C(=O)O)CCc1ccccc1)/CCC=C(C)C	25135579	-10.9
Armofrutin-3	COc1cc(CCc2ccccc2)c(c(c1C[C@@H](C(=C)C)O)O)C(=O)O	38354854	-10.8
Araphyn-4	O[C@H](C(O)(C)C)Cc1c(O)cc(cc1O)/C=C/c1ccccc1	-	-11.6
Araphyn-3	Oc1ccc(cc1)/C=C/c1cc(O)c(c(c1)O)C[C@@H](C(O)(C)C)O	-	-11.3
Araphyn-1	CC(/C=C/c1c(O)cc(cc1O)/C=C/c1ccccc1)C	25187674	-11.9
Arachidin-MeBu	CC[C@H](C(=O)Oc1cc(/C=C/c2ccccc2)c(c(c1/C=C/C(C)C)O)C(=O)O)C	-	-12.9
Arachidin-ival	CC(/C=C/c1c(OC(=O)CC(C)C)cc(c(c1O)C(=O)O)/C=C/c1ccccc1)C	-	-10.4
Arachidin-Bu	CC(/C=C/c1c(OC(=O)C(C)C)cc(c(c1O)C(=O)O)/C=C/c1ccccc1)C	-	-10.3
Arachidin-ang	C/C=C(\C(=O)Oc1cc(/C=C/c2ccccc2)c(c(c1/C=C/C(C)C)O)C(=O)O)/C	-	-10.5
Arachidin-57b	Oc1ccc(cc1)/C=C/c1cc(O)c2c(c1)O[C@@](C)(O)CC=C2	-	-12.1
Arachidin-57ab	Oc1ccc(cc1)/C=C/c1cc2O[C@@](C)(O)CC=Cc2c(c1C(=O)O)O	-	-11.5
Arachidin-57a	OC(=O)c1c(/C=C/c2ccccc2)cc2c(c1O)C=CC[C@](O2)(C)O	-	-11.5
Arachidin-56b	CC(=CCC[C@@]1(C)CCc2c(O1)cc(cc2O)/C=C/c1ccccc1)C	-	-13.2

Table S1: Virtual Screening using Autodock Vina (Part 4)

Molecule	SMILES	CID/C_id	Score/Vina (kcal/mol)
Arachidin-56	Oc1cc(/C=C/c2ccccc2)cc2c1CCC(O2)(C)C	101193354	-11.6
Arachidin-3	CC(/C=C/c1c(O)cc(cc1O)/C=C/c1ccc(cc1)O)C	11380920	-12.3
Anthopogocyclolic-acid	OC(=O)c1c(O)cc2c(c1C)[C@H]1[C@H]3[C@](O2)(C)CC[C@H]3C1(C)C	54753488	-10.1
anhydroCannabimovone	CCCCCc1cc2O[C@H]3[C@H](c2c(c1)O)[C@H](C[C@@H]3C(=O)C)C(=C)C	-	-10.5
Anadamide	CCCCC/C=C\C/C=C\C/C=C\C/C=C\CCCC(=O)NCCO	5281969	-8.4
Amorfrutin-A	COc1cc(CCc2ccccc2)c(c(c1CC=C(C)C)O)C(=O)O	17950432	-11.1
Cannabidiolic_acid-M3	C[C@H](CCCc1cc(O)c(c(c1C(=O)O)O)[C@@H]1C=C(CC[C@H]1C(=C)C)C(=O)O)O	-	-9.8
Cannabidiolic-acid-M2	CCCCCc1cc(O)c(c(c1C(=O)O)O)[C@@H]1C=C(CC[C@H]1C(=C)C)C(=O)O	137518579	-9.8
Canabidiolic-acid-M1	CCCCCc1cc(O)c(c(c1C(=O)O)O)[C@@H]1C=C(CO)CC[C@H]1C(=C)C	137518582	-9.1
Canabidiolic-acid	CCCCCc1cc(O)c(c(c1C(=O)O)O)[C@@H]1C=C(C)CC[C@H]1C(=C)C	160570	-11.7
Canabichromenic-acid-M1	C[C@@H](CCCc1cc2O[C@@](C)(CCC=C(C)C)C=Cc2c(c1C(=O)O)O)O	-	-9.7
Canabichromenic-acid	CCCCCc1cc2O[C@@](C)(CCC=C(C)C)C=Cc2c(c1C(=O)O)O	92448135	-8.9
Acetylcannabigeroquinol	CCCCCC1=CC(=O)C(=C(C1=O)OC(=O)C)C/C=C(/CCC=C(C)C)\C	25172436	-7.8
Acetyl-Abnormal-hydrocannabigeroquinol	CCCCCC1=C(C/C=C(/CCC=C(C)C)\C)[C@H](C=C([C@H]1O[C@H](O)C)O)O	-	-8.3
Abnormal-cannabigeroquinol	CCCCCC1=C(C/C=C(/CCC=C(C)C)\C)C(=O)C=C(C1=O)O	44139743	-9.1
Abnormal-cannabigerol	CCCCCC1=C(C/C=C(/CCC=C(C)C)\C)[C@H](C=C(C1)O)O	-	-8.3
84	CCCCNc1cc(C)c2c(c1)OC=C1[C@@H]2CC(=CC1)C	-	-10.6
83	CC(=CCc1c(CCc2ccccc2)cc(c(c1O)O)O)C	86056178	-12
82	COc1cc(CCc2ccc(cc2)O)ccc1CC=C(C)C	86056215	-13.2
79	C/C(=C\CCc1c(O)cc(c(c1O)C(=O)Cc1ccccc1)O)/CCC=C(C)C	-	-10.9
72d	CC(=C)C(=O)CCC[C@]1(C)CCc2c(O1)cc(c(c2/C=C/c1ccccc1)C(=O)O)O	-	-10.5
72c	CC(=C)[C@H](CCC[C@]1(C)CCc2c(O1)cc(c(c2/C=C/c1ccccc1)C(=O)O)O)O	-	-12.6
72b	OC(=O)c1c(O)cc2c(c1/C=C/c1ccccc1)CC[C@@](O2)(C)CC/C=C/C(O)(C)C	-	-9.4
72a	CC(=CCCC[C@]1(C)CCc2c(O1)cc(c(c2/C=C/c1ccccc1)C(=O)O)O)C	-	-9.2
71b	OC(=O)c1c(O)cc2c(c1/C=C/c1ccccc1)[C@H]1[C@@H]3[C@@](O2)(C)CC[C@@H]3C1(C)C	-	-10.9
71a	Oc1cc(/C=C/c2ccccc2)c2c(c1)O[C@]1([C@H]3[C@H]2C([C@H]3CC1)(C)C)C	-	-11.2
70c	CC(=CCC[C@]1(C)C=Cc2c(O1)cc(c(c2/C=C/c1ccccc1)C(=O)O)O)C	-	-9.5
70b	CC(=CCC[C@]1(C)C=Cc2c(O1)cc(cc2/C=C/c1ccccc1)O)C	-	-8.8
70a	Oc1cc(/C=C/c2ccccc2)c2c(c1)OC(C=C2)(C)C	-	-10.9
69c	CC(=CCC[C@@]1(C)C=Cc2c(O1)cc(cc2O)/C=C/c1ccccc1)C	-	-13.1
69b	OC(=O)c1c(/C=C/c2ccccc2)cc2c(c1O)C=CC(O2)(C)C	-	-12.5
69a	Oc1cc(/C=C/c2ccccc2)cc2c1C=CC(O2)(C)C	101472763	-12.2
68	CC(=C)[C@H]1Oc2c(C1)c(/C=C/c1ccccc1)cc(c2)O	-	-11.5
67	Oc1cc(/C=C/c2ccccc2)c2c(c1)occ2	71453429	-10.9
66	Oc1cc(/C=C/c2ccccc2)c2c(c1)OC([C@@H](C2)O)(C)C	-	-11.3
65d	COc1cc(O)cc(c1CC=C(C)C)/C=C/c1ccc(c(c1)OC)O	66575864	-13.8
65c	COc1cc(O)cc(c1CC=C(C)C)/C=C/c1ccc(c(c1)O)O	71468710	-13.3
65b	COc1cc(/C=C/c2cc(O)cc(c2CC=C(C)C)O)ccc1O	57387613	-13.5
65a	CC(=CCc1c(/C=C/c2ccc(c(c2)O)O)cc(cc1O)O)C	57387612	-13
64	Oc1cc(/C=C/c2ccccc2)c(c(c1)O)C[C@@H](C(=C)C)O	-	-11
63h	CC(=CCc1c(/C=C/c2ccc(cc2)O)cc(cc1O)O)C	129844396	-11
63g	COC(=O)c1c(O)cc(c(c1/C=C/c1ccccc1)C/C=C(\CCC=C(C)C)/C)O	-	-10.5
63f	COc1cc(O)cc(c1C/C=C(\CCC=C(C)C)/C)/C=C/c1ccccc1	-	-9.8
63e	C/C(=C/Cc1c(O)cc(cc1/C=C/c1ccccc1)O)/CCC=C(C)C	-	-10.8
63d	COC(=O)c1c(O)cc(c(c1/C=C/c1ccccc1)CC=C(C)C)O	-	-11.4
63c	COc1cc(/C=C/c2ccccc2)c(c(c1)O)CC=C(C)C	18546453	-10.2
63b	COc1cc(O)cc(c1CC=C(C)C)/C=C/c1ccccc1	6446720	-11.6
63a	CC(=CCc1c(/C=C/c2ccccc2)cc(cc1O)O)C	101193350	-12.1
12-acetoxy-delta-8-Tetrahydrocannabinoic-acid	CCCCCc1cc2OC(C)(C)[C@H]3[C@H](c2c(c1C(=O)O)O)[C@@H](OC(=O)C)C(=CC3)C	-	-11.9
11-hydroxy-9-oxo-delta-8-Tetrahydrocannabinol	CCCCCc1cc(O)c2c(c1)OC([C@H]1[C@]2(O)C(=O)C(=CC1)C)(C)C	-	-11.2
11-acetoxy-delta-8-Tetrahydrocannabinoic-acid	CCCCCc1cc2OC(C)(C)[C@@H]3[C@@H](c2c(c1C(=O)O)O)CC(=C(C3)OC(=O)C)C	-	-9.2
10-Oxo-delta-6a-tetrahydrocannabinol	CCCCCc1cc(O)c2c(c1)OC(C1=C2C(=O)[C@H](C)CC1)(C)C	162394589	-10.7
10-hydroxy-delta-8-Tetrahydrocannabinol-acid	CCCCCc1cc(O)c2c(c1)OC([C@@H]1[C@@H]2CC(=C(C1)O)C)(C)C	-	-9.6
8-Oxo-D9-trans-tetrahydrocannabinol	CCCCCc1cc(O)c2c(c1)OC([C@H]1[C@H]2C=C(C)C(=O)C1)(C)C	101152951	-10.6
8-hydroxyisocannabichromene	CCCCCc1cc2O[C@](C)(CC[C@@H](C(=C)C)O)C=Cc2c(c1)O	-	-10.3
8-Hydroxy-delta(9)-tetrahydrocannabinol	CCCCCc1cc(O)c2c(c1)OC([C@H]1[C@H]2C=C(C)[C@@H](C1)O)(C)C	169652	-11.5
8-hydroxycannabinolic-acid-A	CCCCCc1cc2OC(C)(C)c3c(c2c(c1C(=O)O)O)cc(c(c3)O)C	44139742	-10.8
8-Hydroxycannabinol	CCCCCc1cc(O)c2c(c1)OC(c1c2cc(C)c(c1)O)(C)C	44241652	-9.9
8a-Hydroxy-D9-trans-tetrahydrocannabinol	CCCCCc1cc(O)c2c(c1)OC([C@H]1[C@H]2C=C(C)[C@H](C1)O)(C)C	23620581	-10.5
7-hydroxycannabinol	CCCCCc1cc(O)c2c(c1)OC(c1c2cc(C)cc1O)(C)C	-	-12.4
6-Metiletrapterol-A	Cc1ccc2c(c1)c1c(O)cc(cc1OC2(C)C)[C@H]1COc2c(C1=O)c(O)c(c(c2)O)C	-	-13.9
6ar-Cannabichromanone-C	CCCCCc1cc(O)c2c(c1)OC([C@@H](C2=O)CC(=O)C(=O)C)(C)C	-	-12.8
6ar-Cannabichromanone-B	CCCCCc1cc(O)c2c(c1)OC([C@@H](C2=O)[C@@H](CC(=O)C)O)(C)C	-	-11.6

Table S1: Virtual Screening using Autodock Vina (Part 5)

Molecule	SMILES	CID/C_id	Score/Vina (kcal/mol)
6a-7-10a-trihydroxy-D9-tetrahydrocannabinol	CCCCCc1cc(O)c2c(c1)OC([C@@]1([C@]2(O)C=C(C)C[C@H]1O)O)(C)C	-	-10.4
5-acetyl-4-hydroxycannabigerol	CCCCCc1cc(O)c(c(c1O)OC(=O)C)C/C=C(/CCC=C(C)C)\C	44139615	-8.8
4-Terpinyl-D9-trans-Tetrahydrocannabinolate	CCCCCc1cc2OC(C)(C)[C@H]3[C@H](c2c(c1C(=O)O[C@@]1(CCC(=CC1)C)C(C)C)O)C=C(CC3)C	162889905	-9.8
Cannabiorcol	CCCCCc1cc2OC(C)(C)c3c(c2c(c1C(=O)O[C@@]1(CCC(=CC1)C)C(C)C)O)cc(cc3)C	162847257	-10.7
4-Acetoxycannabichromene	CCCCCc1cc(O)c2c(c1OC(=O)C)O[C@](C=C2)(C)CCC=C(C)C	162952316	-9
2-hydroxy-1-2-dihydrocannabichromene	CCCCCc1cc2O[C@](C)(CCC=C(C)C)[C@H](Cc2c(c1)O)O	-	-10.7
2-Formyl-D9-trans-tetrahydrocannabinol	CCCCCc1cc2OC(C)(C)[C@H]3[C@H](c2c(c1CO)O)C=C(CC3)C	172516719	-10.6
2-araquinoilglicerol	CCCCC/C=C\C/C=C\C/C=C\C/C=C\CCCC(=O)OC(CO)CO	5282280	-8

Table S2: Classes and Subclasses of cannabinoids and cannabinoids-like molecules studied.

Molecules	Plant	Class	Subclass
CMG; R6E	*Cannabis sativa*[100,101]	Cannabinoid	CBG derivate
GLDC	*Glycyrrhiza lepidota*[102]	Stilbenoid	-
OMCNB; CNBA	*Cannabis sativa*[101]	Cannabinoid	CBN derivate
PRT	*Radula perrotteti*[103]; *Radula marginata*[59]	bibenzyl cannabinoid	Cannabinoid-like
THC; D9CT; THCP	*Cannabis sativa*[101]	Cannabinoid	THC derivate
THCVM2	*Cannabis sativa*[104]	Cannabinoid	THCV metabolite
HHC	*Helichrysum umbraculigerum*[105]	CBG derivate	Cannabinoid-like
CNBD	*Cannabis sativa*[105]	Cannabinoid	CNBD derivate
ARAC1; ARAC2	*Arachis hypogea*[106]	Monomeric stilbene	-
MCRA; MCDC; MCDD	*Machaerium Pers*[107]	CBD, THC analogues	Cannabinoid-like
RDLA; RDLK; RDLI; RDLH; RDLJ; RDL59	*Radula buccinifera*; *Radula javanica*;*Radula complanata*[108]	Prenyl bibenzyls	-
DCBA; DDAA	*Glycyrrhiza foetida*[109-111]	Stilbenoid	Cannabinoid-like
DMDD; DMDE	*Desmodium canum*[112]	Isoflavonones	Cannabinoid-like
CNBRPL; HD11	*Cannabis sativa*[101]	Cannabinoid	Miscellaneous
TCNT	*Cannabis sativa*[101]	Cannabinoid	CBT derivate

Table S3: Frontier molecular orbital energies. Values are in eV.

Molecule	HOMO-4	HOMO-3	HOMO-2	HOMO-1	HOMO	LUMO	LUMO+1	LUMO+2	LUMO+3	LUMO+4
ACh	-10.73	-10.10	-10.08	-8.85	-8.22	-0.47	0.91	0.36	0.67	0.77
GNT	-8.02	-7.83	-7.33	-6.85	-6.28	-0.95	-0.44	-0.13	0.00	0.27
DNP	-7.54	-7.33	-7.15	-6.98	-6.15	-1.74	-0.88	-0.59	-0.45	-0.39
LADO	-7.62	-7.51	-7.31	-6.99	-6.62	-1.24	-0.82	-0.57	-0.41	-0.31
RIVA	-9.48	-9.02	-8.29	-7.28	-7.09	-0.85	-0.70	0.02	0.32	0.40
PHYSO	-8.38	-8.16	-7.44	-7.16	-5.87	-0.72	-0.26	0.08	0.25	0.47
THA	-8.97	-8.93	-7.58	-7.30	-6.44	-2.06	-0.99	-0.32	-0.25	0.05
DOP	-9.21	-8.81	-6.68	-6.06	-5.84	-0.40	0.07	0.13	0.32	0.64
HUP	-7.62	-6.89	-6.57	-6.49	-6.10	-1.31	-0.06	0.11	0.20	0.36
HUX	-8.25	-7.73	-7.30	-6.79	-6.55	-2.30	-1.34	-0.62	-0.12	0.01
HUW	-7.69	-7.50	-7.31	-6.63	-6.54	-2.22	-1.19	-0.51	-0.42	-0.40
CMG	-7.74	-7.63	-6.64	-6.23	-6.11	-0.22	-0.01	0.06	0.20	0.35
CNBRPL	-7.94	-7.63	-7.45	-6.27	-6.00	-0.24	0.02	0.04	0.35	0.46
GLDC	-7.02	-7.00	-6.80	-6.28	-6.13	-0.54	-0.42	-0.19	-0.02	0.05
OMCNB	-7.69	-7.54	-6.73	-6.44	-5.91	-1.23	-0.40	-0.16	0.15	0.38
PRT	-7.01	-6.80	-6.35	-6.23	-6.00	-0.50	-0.42	-0.10	0.02	0.06
THC	-7.94	-7.91	-6.46	-5.95	-5.95	-0.19	0.06	0.10	0.37	0.41
THCP	-7.96	-7.87	-6.45	-6.18	-5.97	-0.21	0.04	0.10	0.36	0.42
THCVM2	-8.08	-8.03	-7.29	-6.32	-6.03	-1.54	-0.24	0.00	0.03	0.35
CNBA	-8.13	-7.75	-6.84	-6.38	-6.06	-1.34	-1.02	-0.54	0.02	0.12
HHC	-6.73	-6.44	-6.37	-6.28	-6.19	-1.12	-0.52	-0.31	-0.12	0.00
CNBD	-7.26	-7.09	-6.54	-6.35	-6.16	-0.82	-0.53	-0.15	0.02	0.04
ARAC1	-7.56	-6.76	-6.31	-6.23	-5.43	-1.75	-0.51	-0.17	-0.03	0.04
ARAC2	-7.22	-6.79	-6.47	-6.20	-5.52	-1.60	-0.56	-0.03	0.00	0.15
MCDC	-7.03	-6.77	-6.70	-6.22	-5.78	-1.55	-0.31	-0.10	-0.03	0.11
MCDD	-7.40	-6.98	-6.75	-6.15	-5.78	-1.54	-0.30	-0.13	0.08	0.16
RDLA	-7.02	-6.84	-6.61	-6.49	-6.24	-0.55	-0.42	-0.25	-0.07	0.03
RDLK	-7.79	-7.21	-6.75	-6.14	-5.54	-1.59	-0.56	-0.07	0.08	0.16
RDLI	-7.69	-7.11	-6.79	-6.24	-5.63	-1.72	-0.48	-0.15	-0.03	0.14
RDLH	-7.03	-6.92	-6.78	-6.63	-6.43	-1.72	-0.45	-0.39	-0.21	-0.02
RDLJ	-7.62	-7.11	-6.78	-6.23	-5.65	-1.75	-0.48	-0.09	0.03	0.19
RDL59	-7.01	-6.97	-6.75	-6.69	-6.59	-1.49	-0.70	-0.42	-0.34	-0.01
DCBA	-7.01	-6.81	-6.65	-6.21	-6.09	-0.52	-0.42	-0.17	0.06	0.14
DMDD	-7.16	-6.68	-6.37	-6.24	-6.09	-1.87	-0.48	-0.15	-0.08	0.04
DMDE	-6.68	-6.46	-6.42	-6.25	-6.07	-1.86	-0.39	-0.14	-0.06	-0.01
D9CT	-7.98	-7.85	-6.44	-6.21	-5.97	-0.17	0.03	0.09	0.31	0.47
TCNT	-7.92	-7.76	-7.43	-6.16	-5.78	-0.95	-0.04	0.04	0.35	0.43
HD11	-8.02	-7.61	-6.92	-6.22	-5.99	-0.31	-0.13	0.06	0.20	0.40
DDAA	-7.02	-6.81	-6.57	-6.22	-6.13	-0.52	-0.42	-0.14	0.01	0.05
MCRA	-7.64	-7.11	-6.88	-6.08	-5.69	-1.70	-0.48	-0.03	0.03	0.12
R6E	-8.08	-7.56	-6.39	-6.36	-6.13	-0.26	0.02	0.04	0.32	0.37
Maximum	-6.68	-6.44	-6.31	-5.95	-5.43	-0.17	0.06	0.1	0.37	0.47
Minimum	-8.13	-8.03	-7.45	-6.69	-6.59	-1.87	-1.02	-0.54	-0.34	-0.02
Difference	1.45	1.59	1.14	0.74	1.16	1.7	1.08	0.64	0.71	0.49

*Maximum and Minimum in relation to the cannabinoids and cannabinoids-like molecules; The difference is between maximum and minimum values.

Table S4: Electronic descriptors: Charge on H1, charge on the heteroatom, H1-H2 distance between two most acidic hydrogens, molecular size and volume.

Molecule	H1 (a.u.)	Charge Heteroatom (a.u.)	H1-H2 (Å)	Molecular size (Å)	Volume (cm^3/mol)
ACh	0.19	-0.73	1.80	9.00	198.28
GNT	0.46	-0.84	8.26	9.49	390.58
DNP	0.21	0.34	2.29	16.28	310.53
LADO	0.44	-1.07	6.49	11.97	260.65
RIVA	0.37	-0.73	2.00	11.24	543.29
PHYSO	0.38	-0.79	9.03	12.71	406.67
THA	0.44	-0.81	5.72	9.56	230.46
DOP	0.50	-1.39	3.11	9.25	203.03
HUP	0.43	-1.25	1.62	9.29	282.86
HUX	0.48	-0.80	5.75	11.21	364.39
HUW	0.45	-0.88	5.72	12.30	211.65
CMG	0.50	-0.89	10.36	17.24	337.47
CNBRPL	0.50	-0.90	5.18	15.69	259.95
GLDC	0.44	-0.70	5.65	16.27	248.74
OMCNB	0.20	-0.64	4.30	15.27	213.79
PRT	0.48	-0.70	2.23	15.86	317.08
THC	0.49	-0.73	3.03	15.37	223.41
THCP	0.49	-0.73	3.09	17.88	221.94
THCVM2	0.49	-0.71	4.13	13.17	257.97
CNBA	0.49	-0.73	5.10	15.26	319.58
HHC	0.55	-0.75	12.42	20.17	383.10
CNBD	0.51	-0.77	5.63	15.58	246.28
ARAC1	0.50	-0.69	3.14	16.78	225.19
ARAC2	0.54	-0.76	12.28	16.10	251.56
MCDC	0.50	-0.73	5.70	15.14	313.87
MCDD	0.49	-0.85	7.73	15.97	254.45
RDLA	0.47	-0.68	2.23	14.60	226.64
RDLK	0.49	-0.85	15.11	15.11	234.33
RDLI	0.48	-0.71	3.49	14.62	224.01
RDLH	0.52	-0.74	3.79	14.21	225.95
RDLJ	0.22	-0.57	4.62	14.55	230.98
RDL59	0.52	-0.80	6.57	14.14	203.76
DCBA	0.45	-0.73	5.67	15.38	258.91
DMDD	0.52	-0.75	5.61	16.14	357.30
DMDE	0.52	-0.76	5.62	16.12	343.48
D9CT	0.47	-0.68	2.20	15.35	262.35
TCNT	0.48	-0.87	4.35	15.03	255.40
HD11	0.48	-0.80	4.26	14.76	278.61
DDAA	0.56	-0.78	5.65	15.63	219.66
MCRA	0.49	-0.72	2.20	15.92	308.93
R6E	0.53	-0.75	5.66	13.62	246.50

[a] H1 is the most acidic hydrogen. H1-H2 is the smallest distance between the most acidic hydrogens.

Table S5: General descriptors: Molecular weight (Mw),number of aromatic rings, the fraction of sp3 carbon atoms, rotatable bonds, hydrogen bond acceptors, and hydrogen bond donors.

Molecule	MW (g/mol)	Num Aromatic Rings	Fraction Csp3	Rotatable bonds	H-bond acceptors	H-bond donors
ACh	146.21	0	0.86	4	2	0
GNT	287.35	0	0.53	1	4	1
DNP	380.50	2	0.46	6	3	1
LADO	285.45	1	0.44	7	3	3
RIVA	250.34	1	0.50	6	3	0
PHYSO	275.35	1	0.53	3	3	1
THA	198.26	2	0.31	0	1	1
DOP	153.18	1	0.25	2	3	3
HUP	242.32	0	0.40	0	2	2
HUX	298.81	2	0.39	1	1	1
HUW	315.82	2	0.39	2	1	3
CMG	350.49	1	0.62	10	4	4
CNBRPL	348.48	1	0.71	4	4	3
GLDC	298.38	2	0.26	6	3	3
OMCNB	324.46	2	0.45	5	2	0
PRT	346.46	2	0.33	2	2	1
THC	314.46	1	0.62	4	2	1
THCP	342.51	1	0.65	6	2	1
THCVM2	316.39	1	0.53	3	4	2
CNBA	354.44	2	0.41	5	4	2
HHC	410.50	2	0.32	9	5	4
CNBD	310.43	2	0.33	6	2	2
ARAC1	312.36	2	0.16	4	4	4
ARAC2	298.38	2	0.26	5	3	3
MCDC	364.48	2	0.33	3	3	2
MCDD	378.46	3	0.42	1	4	2
RDLA	278.35	2	0.16	2	2	1
RDLK	312.36	2	0.26	2	4	3
RDLI	278.35	2	0.26	2	2	1
RDLH	322.35	2	0.15	3	4	2
RDLJ	292.37	2	0.30	3	2	0
RDL59	322.35	2	0.15	3	4	2
DCBA	296.40	2	0.30	6	2	1
DMDD	436.50	2	0.50	1	6	3
DMDE	436.50	2	0.42	1	6	3
D9CT	314.46	1	0.62	4	2	1
TCNT	332.43	1	0.60	4	4	3
HD11	330.46	1	0.62	4	3	2
DDAA	282.38	2	0.26	5	2	2
MCRA	348.48	2	0.42	2	2	1
R6E	332.48	1	0.62	9	3	2

Table S6: ADMET descriptors of studied molecules using the web platform SwissADME and OSIRIS software [85,86].

Molecule	MR	TPSA Å^2	Consensus Log P	ESOL Log S	BBB	Drugscore	Druglikeness	Score(kcal/mol)
ACh	39.42	26.30	-2.32	2.23	2.00	0.0	0.0	-5.4
GNT	84.05	41.93	1.92	-2.93	1.00	0.91	6.20	-10.5
DNP	116.27	47.56	3.11	-4.82	1.00	0.63	7.29	-11.60
LADO	83.71	53.52	1.44	-2.72	1.00	0.89	2.64	-9.9
RIVA	73.12	32.78	2.34	-2.69	1.00	0.87	1.81	-8.3
PHYSO	84.93	44.81	1.65	-2.57	1.00	0.86	2.19	-9.5
THA	63.58	38.91	2.59	-3.27	1.00	0.16	-7.18	-9.1
DOP	42.97	66.48	0.46	-0.44	1.00	0.28	0.47	-7.4
HUP	72.87	58.88	1.89	-1.60	1.00	0.73	0.27	-9.9
HUX	90.04	38.01	4.09	-4.56	1.00	0.17	0.84	-10.5
HUW	92.16	62.04	2.40	-3.92	1.00	-1.48	0.23	-10.4
CMG	104.80	80.92	4.13	-4.76	2.00	0.30	-10.26	-12.00
CNBRPL	100.74	69.92	3.65	-4.50	1.00	0.32	-8.78	-12.50
GLDC	89.63	60.69	3.62	-4.41	1.00	0.37	-13.73	-11.50
OMCNB	101.56	18.46	5.58	-5.95	2.00	0.18	-12.83	-12.20
PRT	109.09	29.46	5.43	-6.49	2.00	0.12	-3.59	-12.40
THC	97.91	29.46	5.33	-6.11	1.00	0.23	-11.18	-11.40
THCP	107.52	29.46	5.92	-6.82	2.00	0.18	-19.19	-12.30
THCVM2	90.07	66.76	3.72	-5.09	1.00	0.37	-4.49	-11.20
CNBA	104.05	66.76	4.84	-5.95	2.00	0.20	-14.28	-10.90
HHC	121.01	97.99	5.11	-6.45	2.00	0.24	-1.99	-12.60
CNBD	99.98	40.46	5.52	-6.18	1.00	0.22	-20.65	-12.5
ARAC1	94.42	80.92	3.61	-4.76	2.00	0.37	1.12	-13.00
ARAC2	90.49	60.69	4.00	-4.91	1.00	0.41	-1.34	-11.20
MCDC	111.34	53.60	5.38	-6.74	2.00	0.10	-20.43	-12.60
MCDD	110.56	62.83	4.37	-5.65	2.00	0.20	0.95	-11.20
RDLA	87.13	29.46	4.01	-4.62	1.00	0.23	-1.64	-12.9
RDLK	90.82	69.92	3.00	-4.23	1.00	0.31	-0.65	-12.10
RDLI	85.23	29.46	4.04	-4.64	1.00	0.25	-0.48	-12.30
RDLH	94.09	66.76	3.66	-4.82	1.00	0.23	-1.73	-11.10
RDLJ	89.70	18.46	4.43	-4.84	1.00	0.25	0.19	-12.30
RDL59	94.09	66.76	3.52	-4.47	1.00	0.23	-1.73	-13.30
DCBA	92.94	29.46	4.79	-5.26	1.00	0.39	-0.65	-12.60
DMDD	119.89	96.22	04.05	-5.87	2.00	0.16	-1.22	-11.70
DMDE	121.79	96.22	4.17	-5.74	2.00	0.14	-2.16	-11.90
D9CT	97.91	29.46	5.28	-6.11	1.00	0.23	-11.18	-13.00
TCNT	96.22	69.92	3.26	-3.67	1.00	0.14	-12.22	-11.00
HD11	99.07	49.69	4.55	-5.50	1.00	0.27	-14.19	-11.80
DDAA	88.47	40.46	4.42	-5.05	1.00	0.36	-1.90	-11.10
MCRA	109.56	29.46	5.38	-6.32	2.00	0.11	-5.90	-11.30
R6E	101.45	52.99	5.02	-5.37	1.00	0.21	-9.69	-12.00

Table S7: Classification generated by the K-means algorithm for ADMET+ES data.

Molecules	k=4	k=5	k=6
DOP, GNT, HUP, LADO, PHYSO, RIVA, THA	0	0	0
CNBA, CNBD, HHC, MCDC, PRT	1	1	1
CMG, CNBRPL, D9CT, HD11, R6E, TCNT, THC, THCP, THCVM2,	2	2	2
ACh	3	3	3
ARAC1, ARAC2, DMDD, DMDE, MCDD, MCRA, RDL59, RDLH, RDLI, RDLK	1	4	4
DNP, HUW, HUX, RDLJ	1	4	5
DCBA, DDAA, GLDC, OMCNB, RDLA	1	1	5
